# A tubulin binding molecule drives differentiation of acute myeloid leukemia cells

**DOI:** 10.1016/j.isci.2022.104787

**Published:** 2022-07-19

**Authors:** Thomas R. Jackson, Aini Vuorinen, Laia Josa-Culleré, Katrina S. Madden, Daniel Conole, Thomas J. Cogswell, Isabel V.L. Wilkinson, Laura M. Kettyle, Douzi Zhang, Alison O’Mahony, Deanne Gracias, Lorna McCall, Robert Westwood, Georg C. Terstappen, Stephen G. Davies, Edward W. Tate, Graham M. Wynne, Paresh Vyas, Angela J. Russell, Thomas A. Milne

**Affiliations:** 1 MRC Molecular Haematology Unit, MRC Weatherall Institute of Molecular Medicine, NIHR Oxford Biomedical Research Centre Haematology Theme, Radcliffe Department of Medicine, University of Oxford, Oxford, UK; 2 Department of Chemistry, Chemistry Research Laboratory, University of Oxford, Mansfield Road, Oxford OX1 3TA, UK; 3 Department of Chemistry, Molecular Sciences Research Hub, Imperial College London, 82 Wood Lane, London W12 0BZ, UK; 4 Department of Pharmacology, University of Oxford, Mansfield Road, Oxford OX1 3QT, UK; 5 Axis Bioservices, 189 Castleroe Rd, Coleraine, Co. Londonderry BT51 3RP, Northern Ireland; 6 Eurofins Discovery Phenotypic Services, St. Charles, MO 63304 and Burlingame, CA 94010, USA; 7 Discovery Platform at Recursion, 41 S Rio Grande Street, Salt Lake City, UT 84101, USA; 8 Oxstem Ltd, Midland House West Way, Botley, Oxford OX2 0PH, UK

**Keywords:** Chemistry, Biological sciences, Molecular biology, Molecular medicine, Cancer

## Abstract

Despite much progress in developing better drugs, many patients with acute myeloid leukemia (AML) still die within a year of diagnosis. This is partly because it is difficult to identify therapeutic targets that are effective across multiple AML subtypes. One common factor across AML subtypes is the presence of a block in differentiation. Overcoming this block should allow for the identification of therapies that are not dependent on a specific mutation for their efficacy. Here, we used a phenotypic screen to identify compounds that stimulate differentiation in genetically diverse AML cell lines. Lead compounds were shown to decrease tumor burden and to increase survival *in vivo*. Using multiple complementary target deconvolution approaches, these compounds were revealed to be anti-mitotic tubulin disruptors that cause differentiation by inducing a G2-M mitotic arrest. Together, these results reveal a function for tubulin disruptors in causing differentiation of AML cells.

## Highlights

• Cancer treatments usually focus on killing rapidly growing cells
• We focused on differentiating cancer cells independent of the driver mutation
• We identified tubulin as the drug target
• This work illustrates how phenotypic screening can identify unique drug targets

## Graphical abstract

**Figure:**
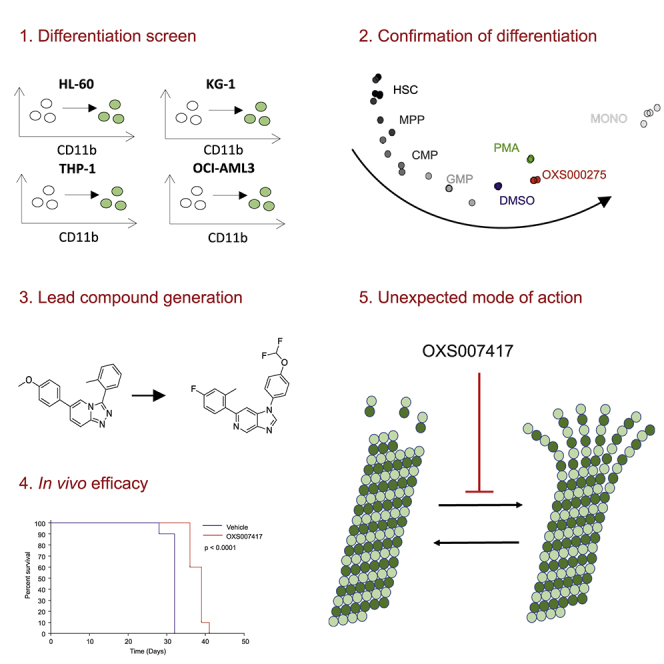

## Introduction

Acute myeloid leukemia (AML) is a hematological malignancy with around 3,000 new cases per year in the UK (Cancer Research UK) and about 21,000 new cases per year in the US (Siegel et al., 2019). AML has a very poor survival rate, especially in elderly patients (5-year survival less than 11% (Daver et al., 2020)) which represent the majority of cases (Cancer Research UK). The current standard of care involves inducing remission using intensive chemotherapy, such as cytarabine in combination with anthracycline-derived antibiotics such as daunorubicin, followed by consolidation chemotherapy or bone marrow transplantation (Daver et al., 2020; Dombret and Gardin, 2016; Yates et al., 1973). Such therapies are not well tolerated by elderly patients and often have extensive side effects (Daver et al., 2020).

The promise of precision medicine is to develop targeted therapies that can specifically impact cancer cells while leaving normal cells unharmed, with the hope that such therapies will be effective and also have fewer toxic side effects. Characterization of a patient’s underlying mutational profile is becoming increasingly important for identifying patient subgroups that will be sensitive to specific targeted therapies. For example, patients that carry mutations in genes such as *fms-like tyrosine kinase 3* (*FLT3*) or *isocitrate dehydrogenase 1* or *2* (*IDH1/2*) can be treated with small molecules designed to specifically target these mutations (Daver et al., 2019; Liu and Gong, 2019). However, because of the high levels of heterogeneity among AML patients (Ivey et al., 2016), even with patients carrying the same driver mutation, often only a subset of patients respond well to targeted therapy, such as in the case of *IDH2* mutations and the drug enasidenib (Bristol Myers Squibb, 2020; Pollyea et al., 2019; Quek et al., 2018; Stein et al., 2019). In general, despite their promise, there has been a high degree of failure in clinical trials for AML with targeted therapies (Cucchi et al., 2021).

Another issue with targeted therapies is that many of them benefit small patient populations only, leaving the wide range of AML patients without effective treatments (Cucchi et al., 2021). The promise of immunotherapies (Jakobsen and Vyas, 2018) and recent exciting clinical trials with the B-cell lymphoma 2 (BCL-2) inhibitor venetoclax (Daver et al., 2020; DiNardo et al., 2018a, 2020; Killock, 2020) represent two approaches for targeting AML not limited to specific mutations. Even with these promising new areas of treatment, there remain patients who do not respond well to treatment. To address this issue, much work is being done on possible combination therapies, although this is often developed empirically without clear underlying mechanistic principles guiding the process (Daver et al., 2020). In all, there continues to be an urgent unmet need for new, well-tolerated therapies that can provide complete durable remission, especially for patient subsets that do not have a clear, well defined molecular target underlying the malignancy (Cucchi et al., 2021; Daver et al., 2020; Watts and Nimer, 2018).

A defining hallmark of AML is a block in the normal myeloid differentiation process, blocking the production of downstream blood lineages and disrupting normal hematopoiesis. An exciting new paradigm in AML treatment is the possibility of inducing normal differentiation of AML cells by removing the differentiation block. Such therapies could be both more effective and less toxic than conventional chemotherapies, and may also provide effective partners for combination therapies. As an exemplar of such an approach, a breakthrough in differentiation therapy was achieved in the treatment of acute promyelocytic leukemia (APL), which represents subset of about 10% of all AML patients (Tallman and Altman, 2008; Wang and Chen, 2008). APL is defined by a specific translocation involving the retinoic acid receptor to create a fusion oncoprotein (Borrow et al., 1990; Dethe et al., 1990) which initially correlated with a poor prognosis. APL is now treatable with an 85% 5-year survival rate (Coombs et al., 2015) because of the introduction of differentiation therapy with all-trans retinoic acid (ATRA); in combination with arsenic trioxide, a compound that causes degradation of the PML-retinoic acid receptor alpha oncogenic driver fusion protein (Miller et al., 2002). However, this therapy targets the specific oncoprotein which represents a vulnerability of APL not found in other AML subsets. Nonetheless, the success of this treatment suggests that the induction of differentiation by other mechanisms could provide different treatments or combination therapies for other subtypes of AML. Indeed, when AML patients carrying *IDH1/2* neomorphic mutations (mIDH, 15–25%) respond to treatment with the specific inhibitors ivosidenib (which targets mIDH1 (Dhillon, 2018)), or enasidenib (which targets mIDH2 (Kim, 2017)), they often display evidence of differentiation (Amatangelo et al., 2017; DiNardo et al., 2018b).

These examples suggest that inducing differentiation in AML may be more effective than current treatments. However, in each of these examples, the drugs have been developed for highly specific targets which are not present in the majority of leukemia patients. Recent work using an *in vitro* screening approach to identify inducers of differentiation resulted in the identification of a class of dihydroorotate dehydrogenase (DHODH) inhibitors. In early preclinical work, DHODH inhibitors appear to be effective at inducing differentiation in AML cells in a non-mutation specific manner (Christian et al., 2019; ClinicalTrials.gov; Sykes et al., 2016). Although it remains unclear how DHODH inhibition directly induces differentiation in AML cells (Christian et al., 2019), this work provides a promising proof of principle that such screening approaches are an effective way of finding compounds for differentiation therapy. However, until the mechanism of action of such compounds is better understood, it is unclear exactly which patient subsets will respond to such a therapy, which could be part of the reason why recent clinical trials for a DHODH inhibitor were terminated because of lack of benefit (ClinicalTrials.gov, 2021). Thus, there remains a further need for the identification of compounds and alternative mechanisms that can induce differentiation in AML cells in a mutation agnostic manner.

Here, we developed an *in vitro* flow cytometry-based phenotypic screen to identify classes of small molecules which are capable of promoting differentiation in AML blasts, and validated their differentiation profiles using RNA-seq. As AML is a highly heterogeneous disease(Papaemmanuil et al., 2016), the phenotypic screen was performed using several AML cell lines to identify molecules whose efficacy was not limited to a particular genetic subtype. From the confirmed hits thus identified, a number of compound series were selected for further optimization. The resulting compounds showed *in vivo* efficacy in reducing tumor burden in a subcutaneous model and displayed increased survival following oral dosing in an orthotopic xenograft model. Using a combination of RNA-seq, BioMAP analysis (an *in vitro* platform which uses primary human cells to test drug efficacy and toxicity) and chemoproteomics, tubulin beta chain was identified as a direct binding target of these compounds. Using other known and structurally distinct tubulin binders, we showed that tubulin disruption causes mitotic arrest, and mitotic arrest results in initiation of differentiation, thus highlighting a mechanism of action and usage for the compounds we have identified.

## Results

### A phenotypic screen for differentiation in multiple AML cell lines identifies several compounds

**Figure 1:**
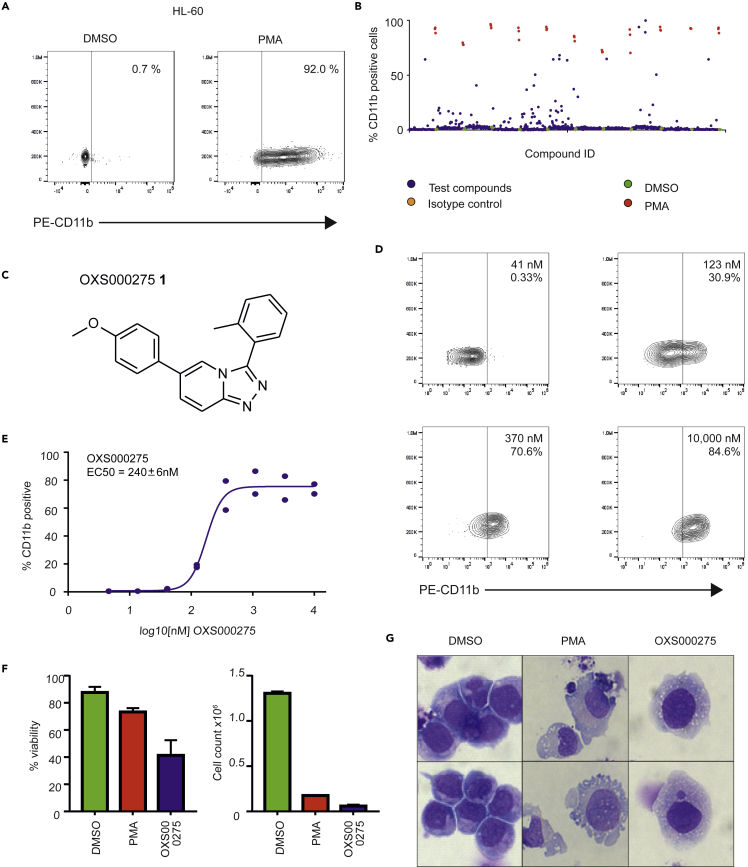
Phenotypic screening and validation of primary hits in HL-60 AML cell line (A) Upon treatment with positive control, PMA (10 nM), HL-60 cells differentiate and up-regulate the cell surface marker CD11b as detected by flow cytometry. (B) Scatterplot distribution showing the results of HL-60 screening of 1000 compound library. (C) Example of a biologically active compound identified (OXS000275 **1**). (D)OXS000275 up-regulated CD11 bat low concentration as shown by flow cytometry. (E) OXS000275 upregulated CD11b in a dose dependent manner with an EC_50_ of 240 ± 6 nM. Two technical replicates were performed per experiment with n = 4 biological replicates. One example curve is shown. EC_50_ value = mean +/- SEM calculated from n = 4 biological replicates. (F) At 4 days post treatment with OXS000275 a reduced viability and cell number was observed. Error bars represent SD of 3 replicates. (G) Cytospin preparations of OX000275-treated or PMA-treated HL-60 cells stained with Wright-Giemsa showed signs of myeloid maturation. See also Figures S1, S2, and Table S1.

A screen was initiated using a library containing 1000 structurally-diverse, commercially available small molecules (See Table S1 for details), in order to identify compounds that were capable of differentiating four AML cell lines (HL-60, OCI-AML3, THP-1, KG-1). Properties of these cell lines are summarized in Table 1, and together they represent approximately 30% of known AML mutations (Papaemmanuil et al., 2016). CD11b is a known cell surface marker of differentiated myeloid cells (DiScipio et al., 1998; Losse et al., 2010) and flow cytometry (FACS) analysis was used to quantify upregulation of CD11b expression after compound treatment. As positive controls, phorbol 12-myristate 13-acetate (PMA (Lee et al., 1991)) was used to induce the differentiation of HL-60 and KG-1 cells (Figures 1A and S1A) whereas tranylcypromine (TCP (Schenk et al., 2012)) was used as a positive control for THP-1 cells and GS87 was used as a positive control for OCI-AML3 cells (Figures S1B and S1C). Cells were treated with 10 μM of compound for 4 days. Compounds that upregulated CD11b more than 10% in at least 3 cell lines were considered potential hits and selected for further investigation. Using this criterion, we identified 44 positive hits (Figures 1B and S1). The structure and CD11b upregulation data of an example hit, OXS000275**1**, is shown in Figures 1C and 1D.

**Table 1:** Cell line properties

Cell Line	Disease	Age	Gender	Source	Molec. Genetics
HL-60	AML M2	35	F	PB	MYC amplification
THP-1	AML M5	1	M	PB	MLL-AF9
KG-1	AML	59	M	BM	Complex Karyo-type
OCI AML3	AML M4	57	M	PB	DNMT3A

### Identified compounds induce both neutrophil and macrophage differentiation in an AML cell line

Concentration-dependent responses were confirmed for hit compounds such as OXS000275, which was found to have a calculated EC_50_ of 240 ± 6 nM (Figures 1D and 1E). Further effects of hit compounds on cell proliferation and viability were measured by staining for dead cells with DAPI and using acridine orange as a counterstain for all cells (Figure 1F). Differentiation was also validated using morphology as characterized by Wright-Giemsa staining, with lighter cytoplasm, a higher cytoplasm to nuclei ratio and an increase in granulation used as signs of differentiation towards a macrophage phenotype (Figure 1G). OXS000275 significantly inhibited cell proliferation and reduced cell viability (Figure 1F) as well as promoting a morphology consistent with differentiation in all four of the cell lines (Figures 1G and S2).

**Figure 2:**
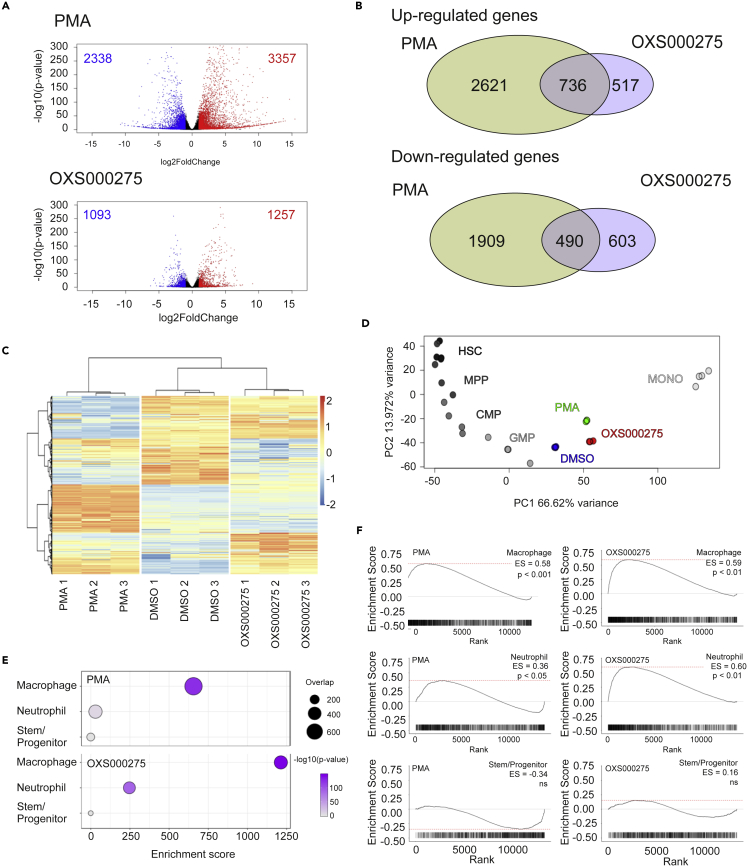
Confirmation of HL-60 differentiation by global gene expression analysis Cells were treated for 3 days with 10 nM of PMA, 1 μM of OXS000275 or 0.1% DMSO. (A) Volcano plot of differentially expressed genes between DMSO control and PMA (top panel) and OXS000275, n=3 samples P-values are calculated from DESeq2. (B) Venn diagram of differentially expressed genes post PMA and OXS000275 treatment. (C) Heatmap of differentially expressed genes. (D) Bulk RNA-sequencing of primary Hematopoietic cells from each population: PCA of each primary Hematopoietic cell population using the top 300 most varied expressed genes: HL-60 cells treated with vehicle (DMSO), PMA or OXS000275 projected onto plot. (E) Treatment with OXS000275 and PMA lead to up regulation of genes consistent with myeloid differentiation when assessed by EnrichR and ARCHS4 Tissues signatures. p values were calculated using Fisher exact test, and Enrichment score as enrichment score = log(p) · z, where z is the *Z* score computed by assessing the deviation from the expected rank. (F) Treatment with OXS000275 and PMA lead to gene-expression changes consistent with myeloid differentiation by gene set enrichment analysis. See also Table S2.

To further confirm that the hits were inducing differentiation, selected compounds were further investigated using RNA-seq analysis, with PMA treatment used as a positive control for differentiation in HL-60 cells. Both PMA and OXS000275 caused genome wide changes in gene expression after 72 h (Figure 2A), but hierarchical clustering of genes showed that OXS000275 and PMA clustered separately from each other and from the DMSO control, demonstrating OXS000275 and PMA caused distinct gene expression profiles (Figure 2B). However, there is a significant overlap between gene expression changes caused by OXS000275 and PMA, suggesting they both modulate common biological processes (Figures 2B and 2C). Using principal component analysis (PCA), compound-treated HL-60 cells were compared to primary human cells of the myeloid lineage (Corces et al., 2016) (Figure 2D). As expected, HL-60s treated with DMSO were found to cluster closer to stem and progenitor cell populations whereas cells treated with PMA and OXS000275 clustered closer to terminally differentiated monocyte populations (Figure 2D). RNA-seq signatures were also analyzed using EnrichR with ARCHS4 signatures, and both PMA and OXS000275 upregulated genes significantly overlapped with those of macrophages, but not stem or progenitor cells (Figure 2E). Despite PMA inducing a larger number of differentially expressed genes, the macrophage signature in PMA treated cells was found to be less significant and produced a lower enrichment score than in OXS000275 treated cells (Figure 2E). This result potentially reflects the promiscuity of PMA and its subsequent impact on a wide range of different biological processes. Besides producing a more specific macrophage signature compared to PMA, OXS000275 treatment also produced a significant neutrophil gene expression profile whereas PMA had little effect on the expression of neutrophil specific genes (Figure 2E). Finally, gene set enrichment analysis (GSEA) confirmed both PMA and OXS000275 signatures were enriched for macrophage genes, whereas OXS000275 was found to also be enriched for genes associated with neutrophils whereas PMA was not (Figure 2F). Taken together these data suggested OXS000275 is able to induce global gene expression changes associated with differentiation at least to the same extent as PMA treatment. However, gene expression changes induced by OXS000275 treatment appeared to be more specific to differentiation than those induced by PMA. Finally, unlike PMA, OXS000275 treatment was able to induce upregulation of both macrophage and neutrophil associated RNA signatures. A summary of EnrichR analysis of other confirmed hits from the phenotypic screen can be found in Table S2.

### Further characterization of lead compounds

**Figure 3:**
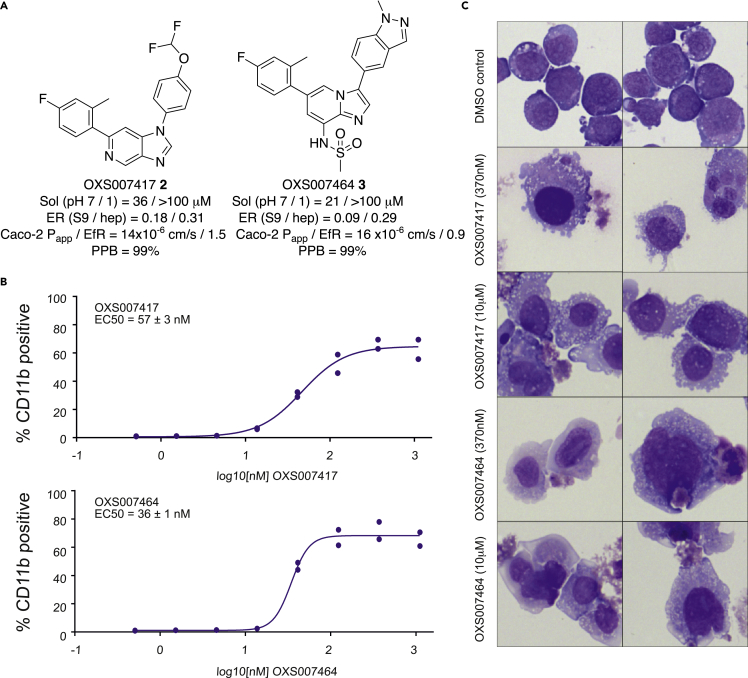
Development of lead compounds (A) Lead compounds OXS007417**2** and OXS007464**3** were developed from original hits. (B) Lead compounds up-regulated CD11b in HL-60 cells by flow cytometry. Two technical replicates were performed per experiment with n = 4 biological replicates. One example curve is shown. EC_50_ values = mean +/- SEM calculated from n = 4 biological replicates. (C) Cytospin preparations of lead compound-treated HL-60 cells stained with Wright-Giemsa showed signs of myeloid maturation. See also Figure S3.

Starting from confirmed hits, further optimization afforded the lead compounds OXS007417**2** and OXS007464**3**, both of which had higher potency and improved ADME properties relative to the starting compound (Figures 3A and 3B, detailed chemistry in (Josa-Cullere et al., 2021)). We confirmed that both OXS007417**2** and OXS007464**3** also caused differentiation of AML cell lines by showing that they upregulated CD11b cell surface expression in HL-60 cells with comparable EC_50_ values of 57 ± 3 nM and 36 ± 1 nM respectively (Figure 3B). Differentiation was further confirmed by morphology as previously described (Figure 3C). In addition, to confirm that OXS007417**2** and OXS007464**3** were causing an increase in the absolute number of CD11b positive cells rather than just selecting for already present CD11b positive cells, we used FACS to quantify the total number of CD11b positive cells in treated wells compared to the DMSO control. The absolute numbers of CD11b positive cells increased (Figure S3), suggesting that the compounds induce true stimulation rather than simple accumulation of differentiated cells.

### Lead compounds demonstrate anti-leukemia activity *in vivo* in a subcutaneous xenograft model of AML

**Figure 4:**
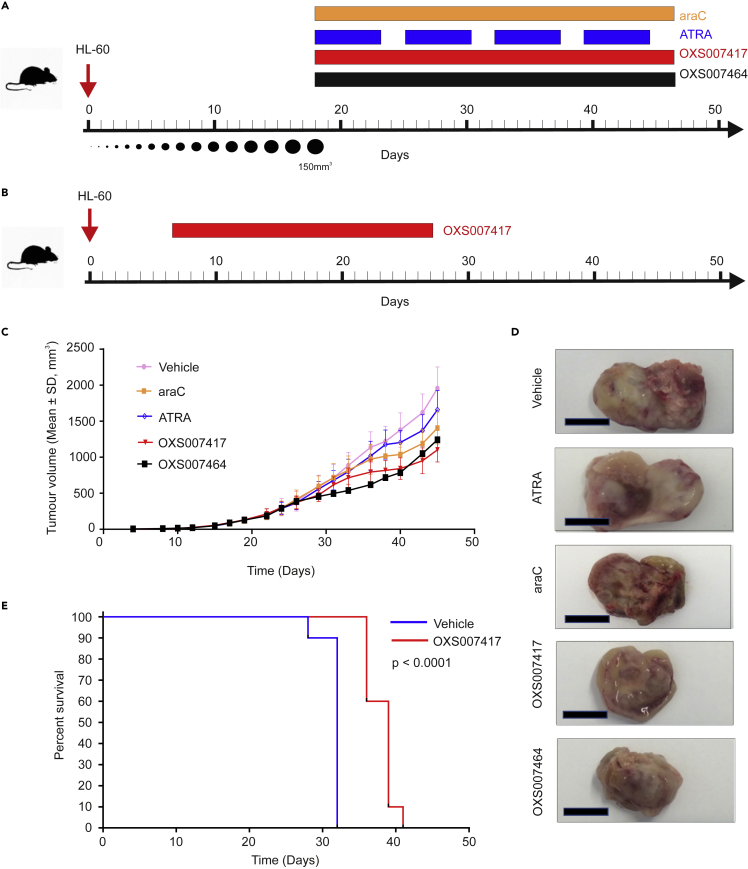
Lead compounds demonstrate anti-leukemia activity *in vivo* in subcutaneous xenograft model and increased survival in orthotopic model (A) Experimental outline of HL-60 subcutaneous xenograft model. Treatment with different compounds began when tumor volume reached 150mm^3^. Specific treatment regimens are detailed in Table 2. (B) Experimental outline of the orthotopic model. Treatment with OXS007417 began 7 days after engraftment. (C) HL-60 cells were implanted subcutaneously onto the flank of female NOD SCID mice, and the mice were treated with vehicle or indicated compounds. Treatment with OXS007417 and OXS007464 reduced tumor growth. Each datapoint represents the mean of n = 10 mice per group, error bars = SD. (D) Example of excised tumors at termination of study; scale bar = 10 mm. (E) OXS007417 prolonged the survival in an orthotopic HL-60 model. NCG mice were engrafted with HL-60 cells. After 7 days, mice were then dosed with OXS007417 bid (formulated in 5% DMSO: 0.1% Tween 20 in PBS; PO; 10 mg/kg, with a dosing volume of 10 mL/kg) for 3 weeks. Survival was analyzed by the Kaplan-Meier method, based on TTE values. The log rank (Mantel-Cox) and Gehan-Breslow-Wilcoxon tests determined the significance of the difference between the overall survival experiences (survival curves) of two groups, based on TTE values. The two-tailed statistical analyses were not adjusted for multiple comparisons, and were conducted at p = 0.05. See also Figure S4.

To study the ability of OXS007417**2** and OXS007464 **3** to inhibit tumor growth *in vivo*, a subcutaneous xenograft model was used in the first instance and compared to standard chemotherapeutic agents. HL-60 cells were implanted into the flank of female NOD SCID mice, and tumors were allowed to reach a volume of 150 mm^3^ before commencement of treatment (Figure 4A). OXS007417 and OXS007464 were administered per os (PO) twice daily for 4weeksat 10 and 3 mg/kg respectively whereas cytarabine (araC) was used as a reference of the standard of care (SoC) for AML (Daver et al., 2020) and administered via intraperitoneal (IP) injection, 20 mg/kg once daily (Altman et al., 2013). Finally, ATRA (PO, 5 mg/kg 5 on/2 off) was used as reference differentiating agent. Treatments are summarized in Figure 4A and Table 2.

**Table 2:** Treatment regimens for subcutaneous model

Group	n	Treatment	Dose Level	Dosing Route	Dosing Regimen	Formulation	Dose volume
1	10	Vehicle	–	PO	BID	5% DMSO and 95% PBS +0.1% Tween 20	10 mL/kg
2	10	AraC	20 mg/kg	IP	QD	sterile water	10 mL/kg
3	10	ATRA	5 mg/kg	PO	5 on/2 off	ethanol 10%; CMC-NA 90%	10 mL/kg
4	10	OXS007417	10 mg/kg	PO	BID	5% DMSO and 95% PBS +0.1% Tween 20	10 mL/kg
5	10	OXS007464	3 mg/kg	PO	BID	5% DMSO and 95% PBS +0.1% Tween 20	10 mL/kg

Treatment with OXS007417 was well-tolerated and did not lead to significant body weight loss in the animals (Figure S4). After 28 days of dosing, OXS007417 significantly delayed the growth of HL-60 tumors, with a tumor control ratio (T/C) of 55%, when compared to vehicle group (p < 0.0001), (Figures 4C and 4D).

The standard of care araC showed a less pronounced effect, with T/C of 70% (p < 0.0001). The smallest effect was observed in the group treated with ATRA, which did not show a significant (p = 0.07) reduction of tumor volume.

At conclusion of the study, plasma and tumor samples were taken for bioanalysis. In animals treated with OXS007417, compound exposure was detected in both samples, at 6.84 × 10^−7^ M (252 ng/mL plasma) and 2.41 × 10^−9^ mol/g (888 ng/g tumor) respectively. In animals treated with OXS007464, compound exposure was detected in both samples, at 3.38 × 10^−7^ M (152 ng/mL plasma) and 4.89 × 10^−10^ mol/g (220 ng/g tumor) respectively. OXS007464 was not as well-tolerated as OXS007417 and the dosage had to be reduced with some mice showing weight loss (Figure S4) and was therefore not used in further *in vivo* studies. The reason for this differing tolerance is unclear.

### Oral administration of OXS007417 improves survival using an *in vivo* murine orthotopic xenograft model of AML

Having shown *in vitro* to *in vivo* correlation using the subcutaneous HL-60 based model, the anti-leukemia activity of OXS007417 was also evaluated in an orthotopic AML model. HL-60 cells were injected into the tail vein of female NCG mice, and 7 days were allowed for engraftment. Animals were then dosed with OXS007417 bid (formulated in 5% DMSO: 0.1% Tween 20 in PBS; PO; 10 mg/kg, with a dosing volume of 10 mL/kg) for 3 weeks (Figure 4B). Significantly, OXS007417 produced prolonged survival (p < 0.0001) compared with the vehicle control group (Figure 4E).

### Analysis from the BioMAP phenotypic platform suggests tubulin disruption as a mechanism of action (MoA) for OXS007417 and OXS007464

**Figure 5:**
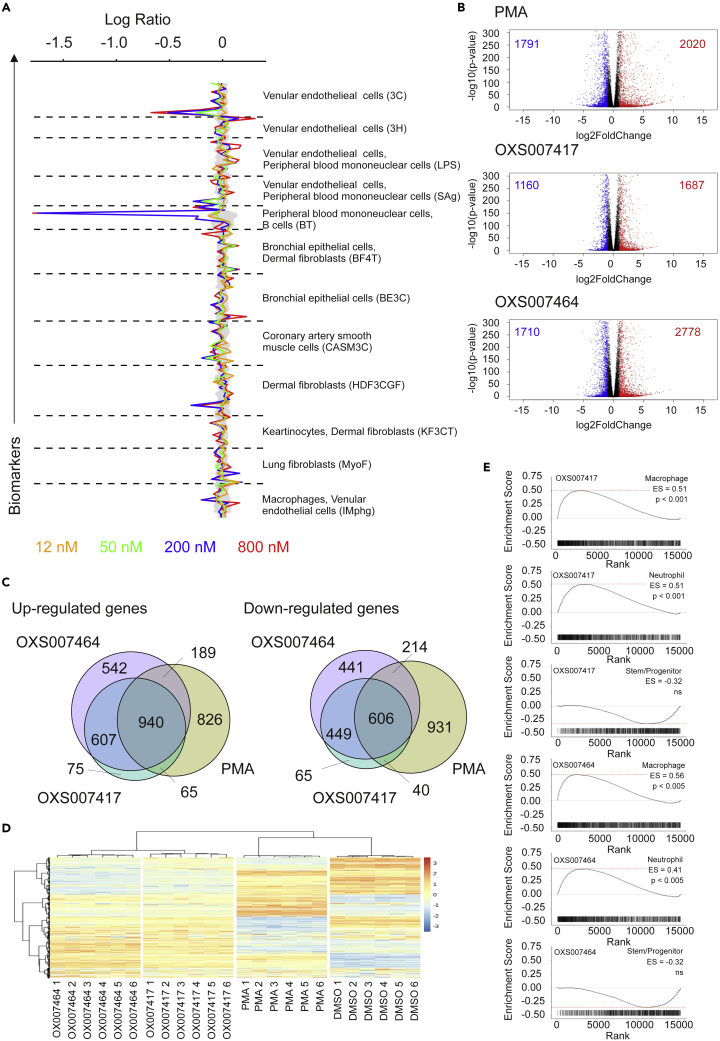
Elucidation of lead compounds mechanisms of action (A) BioMAP analysis of OXS007417 and OXS007464. Primary cell systems created from pooled donors were treated at four indicated concentrations. Cell system specific readouts were taken at time points optimised for each cell system. Readings from treated samples were divided by the average of control readings to generate a ration that was the log_10_ transformed. Significance prediction envelopes (gray) were calculated from control data at 95% confidence intervals. (B) Volcano plot of differentially expressed genes between DMSO control and PMA (top panel), OXS007417 and OXS007464 after a 6 h treatment, n=6 samples P-values are calculated from DESeq2. (C) Venn diagram of differentially expressed genes post PMA, OXS007417 and OXS007464 6 h treatment. (D) Heatmap of differentially expressed genes in six biological replicates treated with either OXS007417, OXS007464, PMA (as a positive control for differentiation) or DMSO (solvent only control) for 6 h. (E) Treatment with OXS007417 and OXS007464 lead to gene-expression changes consistent with myeloid differentiation by gene set enrichment analysis.

With compounds in hand displaying *in vitro* differentiation properties and *in vivo* efficacy in two different tumor models, we next set out to identify the biological targets of the compounds and to identify possible mechanisms of action (MoA) for their activity. The Diversity PLUS panel (BioMAP®, Eurofins Discovery) is an *in vitro* platform which uses different primary human cell types to generate activity profiles designed to aid MoA studies and the identification of off-target effects as well as potential toxicity issues. The method utilizes 12 primary cell-based systems modeling a broad scope of human tissue and disease biology and 148 protein biomarkers to create a “biomarker signature profile” that can be compared to a database of mechanistic signatures for >4600 compounds with known MoAs. The BioMAP study found OXS007417 and OXS007464 to be anti-proliferative to human primary B, T cells, coronary artery smooth muscle cells, endothelial cells, and fibroblasts (Figure 5A), but not cytotoxic at the four concentrations tested (12–800 nM). Comparing the biological activities of OXS007417 and OXS007464 to those of known bioactive agents in the BioMAP reference database, OXS007417 and OXS007464 were found to have profiles most similar to microtubule disruptors (Tables 3 and 4).

**Table 3:** Top 3 similarity matches from an unsupervised search of the BioMAP Reference Database of >4,000 agents for each concentration of OXS007464

Dose	Database match	BioMap Z-standard	Pearson’s Score	# of common biomarkers	Mechanism Class
800 nM	Colchicine 1.1 μM	17.479	0.896	148	Microtubule Disruptor
	Fosbretabulin Disodium 1.1 μM	17.247	8.892	148	Microtubule Disruptor
	Fosbretabulin Disodium 3.3 μM	17.157	0.891	148	Microtubule Disruptor
200 nM	Fosbretabulin Disodium 3.3 μM	19.495	0.924	148	Microtubule Disruptor
	Fosbretabulin Disodium 10 μM	19.257	0.922	147	Microtubule Disruptor
	Fosbretabulin Disodium 30 μM	19.188	0.921	148	Microtubule Disruptor
50 nM	GSK46136A, 370nM	16.977	0.877	148	PLK1 inhibitor
	Vincristine Sulfate, 14 nM	16.926	0.877	148	Microtubule Disruptor
	Pironectin, 14 nM	16.486	0.881	146	Microtubule Disruptor
12 nM	Erastin, 370nM	5.678	0.496	112	VDAC2 Blocker
	SR-2640, 30 μM	4.851	0.392	140	Leukotriene
	Bemegride, 32 μM	4.364	0.428	94	GABA-A Receptor Antagonist

The similarity between agents is determined using a combinatorial approach that accounts for the characteristics of BioMAP profiles by filtering (Tanimoto metric) and ranking (BioMAP Z-Standard) the Pearson’s correlation coefficient between two profiles. Profiles are identified as having mechanistically relevant similarity if the Pearson’s correlation coefficient is ≥0.7.

**Table 4:** Top 3 similarity matches from an unsupervised search of the BioMAP Reference Database of >4,000 agents for each concentration of OXS007417

Dose	Database match	BioMap Z-standard	Pearson’s Score	# of common biomarkers	Mechanism Class
800 nM	Pironetin, 14 nM	18.362	0.911	146	Microtubule Disruptor
	Pironetin, 41 nM	17.424	0.897	146	Microtubule Disruptor
	Epothilone B, 37 nM	17.402	0.895	148	Microtubule Disruptor
200 nM	Pironetin, 14 nM	21.666	0.948	146	Microtubule Disruptor
	Epothilone B, 37 nM	20.508	0.936	148	Microtubule Disruptor
	Epothilone B, 12 nM	19.731	0.927	148	Microtubule Disruptor
50 nM	Paroxetine Hydrochloride, 14 μM	7.380	0.639	98	SERT Antagonist
	Nifedipine, 14 μM	6.934	0.612	98	L-type Ca++ Channel Antagonist
	TMP-153, 370 μM	6.926	0.519	148	ACAT Inhibitor
12 nM	Droperidol, 14 nM	4.935	0.388	148	Dopamine R Antagonist
	Fludrortisone, 14 nM	4.739	0.376	147	GR Agonist
	Sunitinib Malate, 14nM	4.577	0.363	148	VEGFR2 Inhibitor

The similarity between agents is determined using a combinatorial approach that accounts for the characteristics of BioMAP profiles by filtering (Tanimoto metric) and ranking (BioMAP Z-Standard) the Pearson’s correlation coefficient between two profiles. Profiles are identified as having mechanistically relevant similarity if the Pearson’s correlation coefficient is ≥0.7.

### Analysis by early RNA-seq suggests tubulin disruption as a MoA for OXS007417 and OXS007464

To further investigate the MoA of our compounds, HL-60 cells were treated for 6 h with OXS007417**2** (1 μM), OXS007464**3** (1 μM), PMA (10 nM) or DMSO control before global gene expression changes were assessed by RNA-seq (Figures 5B–5E). Even at this early time point, OXS007417, OXS007464 and PMA induced genome wide changes in gene expression (Figures 5B–5D).

As with OXS000275, hierarchical clustering of genes showed that both OXS007417 and OXS007464 had distinct gene expression profiles from PMA and DMSO vehicle control (Figure 5D). Significant overlap of differentially expressed genes with PMA again suggested the activation of common biological processes (Figure 5C). Although forming their own distinct clusters, OXS007417 and OXS007464 clustered together with very similar gene expression profiles compared to each other, relative to PMA and DMSO treatment (Figure 5D). The similarity between OXS007417 and OXS007464 gene signatures is further highlighted by the almost complete overlap of differentially expressed genes caused by these two compounds (Figure 5C). The similarity between OXS007417 and OXS007464 at this early time point suggests a common MoA is shared by these two compounds.

The L1000CDS2 database (Duan et al., 2016) was used to search for substances that can mimic the gene expression changes observed when treating HL-60 cells with PMA, OXS007417 and OXS007464. The top ranked compounds are shown in Table 5. The top ranking matches for our PMA-generated signatures were monopolized by signatures produced by PMA itself or ingenol 3,20-dibenzoate (also a PKC activating compound). Top ranking matches of OXS007417 and OXS007464 were, however, dominated by microtubule disruptors, confirming the BioMAP analysis. In addition, gene set enrichment analysis (GSEA) of OXS007417 and OXS007464 gene signatures confirmed the up-regulation of macrophage and neutrophil differentiation profiles at this time point (Figure 5E).

**Table 5:** Top 14 similarity matches in L1000CDS(Duan et al., 2016) database of 6 h RNA-seq signatures

	PMA			OXS007417			OXS007464		
RANK	Perturbation	1-cos(α)	Cell line	Perturbation	1-cos(α)	Cell Line	Perturbation	1-cos(α)	Cell Line
1	Ingenol 3,20-dibenzoate	0.6076	PL12	CYT997	0.5679	PL21	CYT997	0.5625	PL2
2	PMA	0.632	PL12	PX12	0.5868	PL21	PX12	0.5773	PL2
3	Ingenol 3,20-dibenzoate	0.6407	SKM1	LY-2183240	0.5929	THP1	LY-2183240	0.5866	THP1
4	PMA	0.6433	NOMO1	CYT997	0.5981	THP1	CYT997	0.5982	THP1
5	PX12	0.6581	PL21	ABT-751	0.6034	PL21	ABT-751	0.5993	PL21
6	Ingenol 3,20-dibenzoate	0.6613	HA1E	LY-2183240	0.613	PL21	LY-2183240	0.603	PL21
7	PMA	0.6658	HA1E	PX12	0.6152	THP1	PX12	0.6161	THP1
8	PMA	0.675	SW60	SB225002	0.6159	THP1	ABT-751	0.6199	THP1
9	BRD-k9114395	0.6828	MCF7	ABT-751	0.6192	THP1	SB-225002	0.6249	THP1
10	Ingenol 3,20-dibenzoate	0.6847	SW60	CYT997	0.6274	SKM1	CYT997	0.6298	SKM1
11	CYT997	0.6848	PL21	ABT-751	0.6333	NOMO1	PMA	0.6346	SW620
12	PMA	0.6865	A375	PMA	0.6391	SW620	Ingenol 3, 20-dibenzoate	0.6366	SW620
13	Ingenol 3,20-dibenzoate	0.6881	MDST8	SB225002	0.6395	PL21	ABT-751	0.6379	NOMO1
14	PX12	0.6885	THP1	Ingenol 3, 20-dibenzoate	0.6436	SW620	BRD-K92317137	0.6394	THP1

### Chemoproteomics with photoaffinity labeled probes identifies tubulin as a target for the OXS007464 chemotype

**Figure 6:**
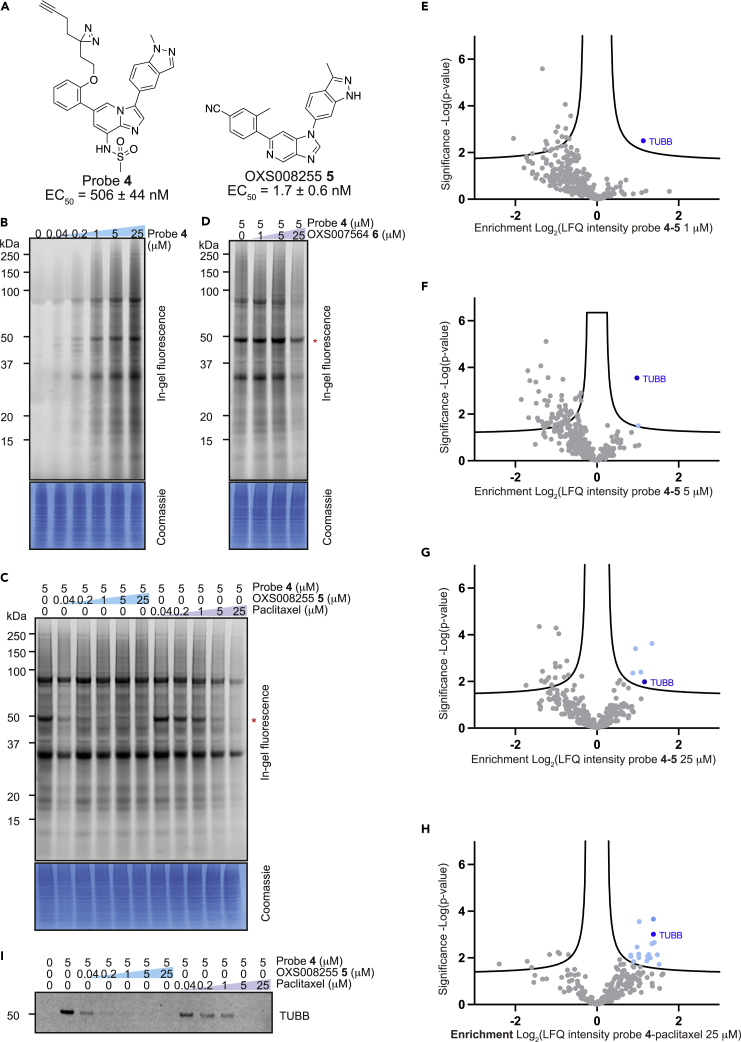
Target identification using chemical probe and chemoproteomics (A) Chemical structures of probe **4** and OXS008255**5**. EC_50_ values for CD11b upregulation are represented as means ± SEM. In-gel fluorescence showing: (B) dose-dependent labelling by probe **4**; (C) competition of OXS008255**5** and paclitaxel with probe **4**; (D) competition of inactive analogue OXS007564**6** with probe **4**. Coomassie stain shows equal protein loading on each gel. Volcano plots showing significantly enriched proteins in the pull-down experiment by probe **4** compared to competition with: (E) 1 μM of OXS008255 **5**; (F) 5 μM of OXS008255**5**; (G) 25 μM of OXS008255**5**; (H) 25 μM of paclitaxel. For (E)-(H) a student’s t-test (FDR = 0.05; S0 = 0.1) was performed between the active probe sample and each DMSO control, and between active probe sample and probe/parent competition samples. Full list of proteins for each volcano plot is available in the supplementary information; (I) Confirmation of tubulin beta chain enrichment by pull-down and immunoblotting. Western blot is cropped, full western blot available in Figure S5. See also Figure S5 and Tables S3, S4–S7, and S8.

Chemoproteomics using clickable photoaffinity labeled probes has proven to be a powerful and useful method for identification of small molecules’ direct target(s) within live cells (Eirich et al., 2012; Li et al., 2018; Parker et al., 2017; Shi et al., 2012; van Delft et al., 2019). Particularly, UV-induced covalent bond formation between a photoaffinity labeled probe and its target(s) has proven especially useful for target identification of small molecules which bind reversibly and with relatively low affinity to their targets through noncovalent interactions (Wilkinson et al., 2020). The clickable alkyne tag enables the Cu(I)-catalyzed azide‒alkyne cycloaddition (CuAAC) reaction with a fluorophore- or biotin-azide allowing visualization and isolation of bound target(s) by pull-down experiments respectively. Thus, on the basis of structure-activity relationship information of the parent compound OXS007464 (Josa-Cullere et al., 2021), we devised an affinity-based protein profiling strategy to elucidate the protein targets of OXS007464 through the design and synthesis of clickable photoaffinity labeled probe **4**, which retained the ability to upregulate CD11b with reasonable levels of potency (EC_50_ = 506 ± 44 nM, Figure 6A).

In order to profile the ability of probe **4** to bind to proteins in cells, probe-treated HL-60 cells were exposed to irradiation (365 nm) and the resulting lysates were treated with TAMRA-azide underCuAAC conditions. Labeled proteins were separated by SDS-PAGE and visualized by in-gel fluorescence. Probe **4** demonstrated clear concentration-dependent labeling of proteins in HL-60 cells (Figure 6B). Furthermore, it was confirmed that labeling is UV-dependent (Figure S5A).

Next, we wanted to distinguish therapeutically relevant target(s) from non-specific binding by carrying out competition control experiments using an excess of more potent parent compounds to block the binding of probe **4**. HL-60 cells were pre-treated with increasing concentrations of parent compounds OXS007464**3** and OXS008255**5** (EC_50_ = 1.7 ± 0.6 nM, Figures 6C and 6D), the latter compound was a result of further optimization of OXS007417. Furthermore, the competition experiment was performed with known tubulin binder paclitaxel (Nogales et al., 1995). Only one band (∼50 kDa, the known molecular weight of tubulin) was clearly competed away in all cases, (Figures 6C and S5A), with OXS008255**5** showing more potent competition than paclitaxel (Figure 6C). Additionally, pre-treatment with OXS007564**6** (Figure S5B), a structurally similar but inactive analogue of OXS007464**3**, had no influence on the binding of probe **4** up to 5 μM (Figure 6D), confirming the importance of the 50 kDa band as a relevant target.

To confirm the identity of the 50 kDa band and to identify other potential targets that may be less abundant and not easy to identify in the gel-based assay, we next performed a proteome-wide pull-down experiment. HL-60 cells were treated with probe **4** alongside DMSO vehicle and competition controls consisting of OXS008255**5** and paclitaxel. After photocrosslinking, the resulting lysates were subjected to CuAAC reaction with biotinylated AzRB capture reagent (Figure S5C) (Broncel et al., 2015). Bound proteins were isolated by using NeutrAvidin beads followed by on-bead digestion and analysis of resulting peptides by nanoLC-MS/MS (full proteomics data available in Tables S3, S4–S7, and S8).

Data analysis revealed that tubulin beta chain (TUBB) was the protein most significantly enriched by probe **4** when compared to DMSO vehicle (Figure S5D). Interestingly, competition experiments with OXS008255 **5** (Figure 6E - 1 μM and 6F - 5 μM) highlighted the high selectivity of our compounds for the tubulin beta chain. Moreover, the competition experiments with 25 μM of OXS008255**5** (Figure 6G) and paclitaxel (Figure 6H) indicated tubulin beta chain and several other proteins as significant, however, tubulin beta chain was the only consistent target across all four conditions. The identification of tubulin beta chain was further confirmed by immunoblotting after the pull-down experiment (Figures 6I and S5E). The results clearly show concentration-dependent competition with OXS008255**5** and paclitaxel confirming tubulin beta chain as a direct target of our compounds.

### OXS007417 disrupts tubulin polymerization in a cell-free system and causes metaphase arrest *in vitro*

**Figure 7:**
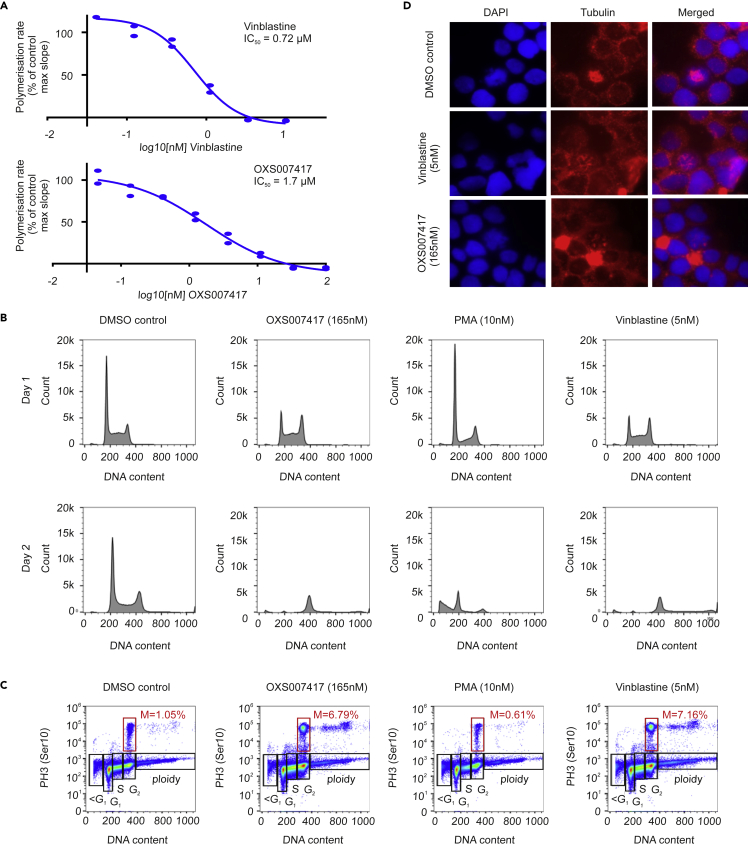
OXS007417 causes mitotic arrest via tubulin disruption in HL-60 cells (A) OXS007417 compared to Vinblastine both disrupt tubulin polymerisation in a cellfree assay. Two replicates, representative curve of 3 biological repeats, EC_50_ value = mean +/- SEM calculated from n=3 biological replicates. (B) Analysis of DNA content by flow cytometry demonstrates G2M arrest in OXS007417 and Vinblastine but not PMA treated HL-60 cells. (C) PH3-staining confirms metaphase arrest upon treatment with OXS007417 and Vinblastine but not PMA. (D) Immunohistochemistry for tubulin (red) and counter staining for DAPI demonstrates spindle disruption by OXS007417 and Vinblastine.

**Figure 8:**
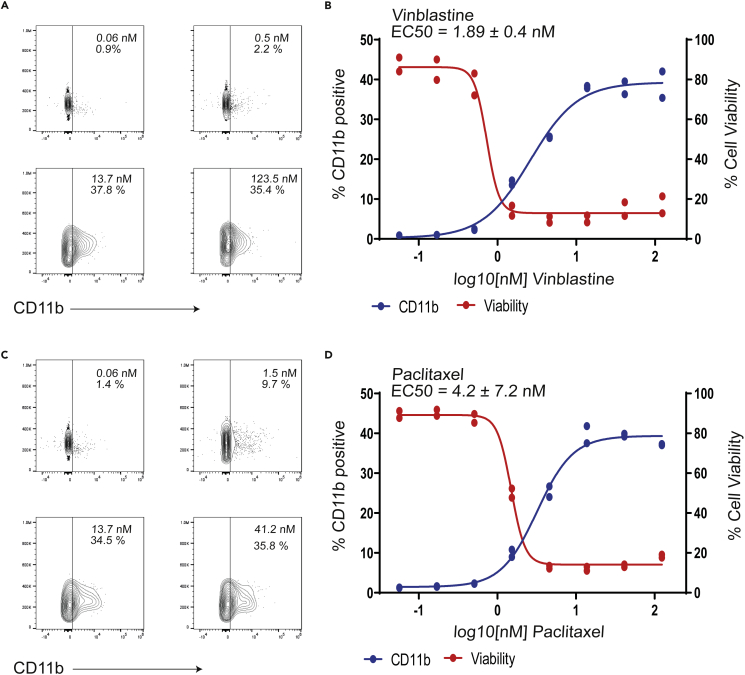
Tubulin disrupters with diverse structures cause up regulation of CD11b (A) Treatment of HL-60 cells with Vinblastine lead to an up-regulation of CD11b in a dose dependent manner when measured by flow cytometry. (B) Dose-response curve of CD11b upregulation and cell viability in response to Vinblastine as determined by flow cytometry. Two technical replicates were performed per experiment with n = 4 biological replicates. One representative example curve is shown. EC_50_ value = mean +/- SEM calculated from n=4 biological replicates. (C) Treatment of HL-60 cells with Paclitaxel lead to an up-regulation of CD11b in a dose dependent manner when measured by flow cytometry. (D) Dose-response curve of CD11b up regulation and cell viability in response to Paclitaxel as determined by flow cytometry. Two technical replicates were performed per experiment with n = 2 biological replicates. One representative example curve is shown. EC_50_ value = mean +/- SEM calculated from n=4 biological replicates. See also Figure S6.

Next, OXS007417 was tested for its ability to inhibit polymerisation of tubulin in a cell-free system. OXS007417 was found to inhibit tubulin polymerization with an IC50 value 1.7 μM for OXS007417, compared to an IC_50_ of 0.72 μM for vinblastine (Figure 7A). The ability of OXS007417 to disrupt the cell cycle of HL-60 cells was subsequently analyzed by DNA and P-H3 staining. Cell cycle analysis showed OXS007417 was able to cause G2-M mitotic arrest with cell cycle profiles comparable to those produced by positive control vinblastine, a known microtubule disruptor (Figures 7B and 7C). Finally, mitotic spindle disruption was observed by immunohistochemistry (Figure 7D). OXS007417 was found to disrupt spindle formation with spindle morphology comparable to that of vinblastine treated cells. To determine if inducing differentiation was a common feature of tubulin disruptors, we tested 6 structurally distinct tubulin disruptors and found they all had varying abilities to upregulate CD11b expression on HL-60 cells, displaying a range of EC_50_ values and an ability to upregulate CD11b to a maximum of about ∼30% (Figures 8A–8D and S6). This suggests that inducing differentiation could be a general feature of tubulin disruption, although cell viability also decreased at the same time (Figures 8A–8D). Together, these results reveal that using an unbiased phenotypic screen, we were able to identify a series of compounds that induce differentiation of four AML cell lines by binding directly to tubulin and causing a G2-M cell-cycle arrest.

## Discussion

Despite the long term need for new therapies in AML, it has only been the last few years that have produced several new therapies approved for use in the clinic (Daver et al., 2020; Patel and Gerber, 2020). However, although these recent advances have been quite exciting, many of these therapies are only able to target a subset of AML patients. In addition, even when new treatments are highly successful in targeting a specific patient subset, there are many individual patients who still do not respond fully to treatment. Thus, there is still a need for drugs that can target patients in a mutation and subtype independent manner, preferably with low toxicity either alone or in combination with other therapies. One promising avenue of research to this end is the identification of compounds such as DHODH inhibitors that can induce differentiation of leukemia cells (Christian et al., 2019; Sykes, 2018). Here, using an unbiased phenotypic screening approach, we have identified a class of tubulin binding molecules and have revealed a mechanism in which tubulin binding can cause a G2-M arrest and differentiation of AML cells.

In order to accomplish this, we used a flow cytometry based phenotypic screen with CD11b as a pan marker of myeloid differentiation to identify compounds that could differentiate several AML cell lines. Differentiation was confirmed by an arrest in cell proliferation, appearance of a differentiated cell morphology and by global gene expression analysis. Thereafter, through iterative rounds of optimization of these hits, a number of lead molecules were developed from initial hits. Using these compounds *in vivo*, efficacy was demonstrated in two different xenograft models.

The advantage of phenotypic screening is that unlike target-based approaches, a well-designed phenotypic screen can directly select for compounds that have the desired cellular activity, in this case differentiation of AML cells. This provides the possibility of selecting for compounds that have polypharmacology as well as identifying compounds that impact desired pathways through distinct mechanisms. It is generally not possible to easily accomplish either of these goals with single target-based approaches, as extensive knowledge of the target is needed to maximize the probability of success and for biomarker purposes. Because of this, it has been argued that successful target-based approaches have often been dependent on previous phenotypic screening for identification and development of first-in-class compounds, and that phenotypic screening is an important complementary approach to target-based methods (Moffat et al., 2017; Swinney, 2013; Vincent et al., 2022).

However, there are several challenges of phenotypic screens including the importance of including counterscreening approaches to filter out compounds which interact with undesirable, undruggable or toxicity inducing targets; as well as identifying the target of a compound and then understanding the underlying mechanism of how target engagement impacts the cell. Here, we used several complementary approaches to identify the direct target of our compounds and to ultimately decipher their MoA. To accomplish this, representative lead compounds were subjected to BioMAP analysis, early time point RNA-seq analysis and chemoproteomics, with all three methods converging on tubulin disruption as the MoA. Furthermore, the compounds were found to upregulate differentiation signatures after just 6 h treatment (Figure 5).

Tubulin inhibitors are well known drugs in cancer therapy (Arnst et al., 2019), but as far as we know they have not previously been identified as compounds that could cause differentiation of cells. More commonly, disruption of tubulin is known to lead to cell death, something we also observe in our data here. Exactly how a G2-M mediated arrest could lead to differentiation is not clear, but interestingly, DHODH inhibitors also seem to have this dual role in promoting a choice between differentiation or cell death (Christian et al., 2019). More specifically, DHODH inhibition induced apoptosis by day 6 of treatment, potentially as an endpoint of differentiation (Christian et al., 2019). One possibility is that AML cells may be prone to differentiating when placed under stress, and it will be important in future work to determine whether other cancer cell types behave in a similar way.

Although tubulin binding drugs such as the taxanes have shown efficacy in the treatment of a range of cancers, their utility is limited predominantly by the emergence of resistance. There is some evidence to suggest resistance can arise because of mutations in β-tubulin, but much more commonly alternative tubulin-independent mechanisms of resistance have been uncovered from analysis of clinical samples (Ben-Hamo et al., 2019; Han et al., 2021; Zhang et al., 2020). These observations have led to renewed interest in the discovery and development of tubulin binding molecules. Further studies with our compounds will be important to determine whether they can circumvent the issues with existing tubulin binders such as the taxanes. There have been two interesting developments in this area that partly validates the possibility of using tubulin binding molecules for AML treatment. Mebendazole, a well-known compound used to treat parasitic worm infections, is also a microtubule disrupting agent that has been repurposed as a potential treatment for various cancers (Pantziarka et al., 2014), most notably as a potential differentiation therapy in AML (Li et al., 2019; Walf-Vorderwulbecke et al., 2018). Interestingly AB8939, a next generation microtubule disruptor from AB Science has recently entered a Phase1/2 study in patients with refractory and relapsed AML (ClinicalTrials.gov, 2022). Together, these observations suggest that there may be potential for pursuing tubulin binding molecules in difficult to treat cancers.

In conclusion, we have shown that phenotypic screening can be employed to identify compounds that exhibit the desired phenotypic activity (differentiation induction) and that a variety of complementary methods can be used in the context of target deconvolution to decipher their MoA. Further work needs to be done to identify other compounds capable of causing differentiation of AML cells, as this is likely to have a long-term impact on cancer therapy, especially in those areas currently lacking safe and effective treatments.

### Limitations of study

The mechanism linking microtubule disruption to differentiation has not been completely addressed, and it is not clear from this study if this is an effect specific to myeloid cells or is more universal. In addition, the article does not contain any docking data. Over 25 binding sites on tubulin have been described. Studies we would like to perform in the future would include competition studies supporting a specific binding mode and binding site on tubulin. Finally, the article was not able to address whether the compounds in this study would behave better than other tubulin binding molecules in AML clinical trials.

## STAR★Methods

### Key resources table

**Table:** 

REAGENT or RESOURCE	SOURCE	IDENTIFIER
**Antibodies**		

CD11b/Mac-1 PE labeled	BD Bioscience	Cat#555388; RRID: AB_395789
anti-TUBB antibody	Invitrogen	Cat#MA5-16308; RRID: AB_2537819
PE Mouse IgG1, κ Iso-type Control	BD Bioscience	Cat# 555749; RRID: AB_396091
anti-mouse secondary antibody (Alexa Fluor™ Plus 800	ThermoFisher	Cat#A32730; RRID: AB_2633279
Alexa Fluor® 488 anti-Histone H3 Phospho (Ser10)	BioLegend	Cat#650804; RRID: AB_10918435

**Chemicals, peptides, and recombinant proteins**		

Protease Inhibitor Cocktail Set III, EDTA-Free - Calbiochem	Merck	Cat#539134
TAMRA-azide	Sigma-Aldrich	Cat#760757
Biotin-PEG3-azide	Sigma-Aldrich	Cat#762024
AzRB	Broncel et al., 2015	N/A
CuSO_4_	Sigma-Aldrich	Cat#451657
TCEP	Sigma-Aldrich	Cat#C4706
TBTA	Sigma-Aldrich	Cat#678937
NuPAGE™ LDS Sample Buffer (4X)	Invitrogen	Cat#NP0007
NuPAGE™ MES SDS Running Buffer (20X)	Invitrogen	Cat#NP0002
NuPAGE™ 4 to 12%, Bis-Tris, 1.0 mm, Mini Protein Gels	Invitrogen	Cat# NP0321BOX
NeutrAvidin agarose resin	Thermo Scientific	Cat#29201
Dynabeads MyOne Streptavidin T1	Thermo Scientific	Cat#65601
Trypsin	Promega	Cat#V5111
2-Chloroacetamide	Sigma-Aldrich	Cat#C0267
DAPI	Sigma-Aldrich	Cat#D9542
Propidium iodide	Sigma-Aldrich	Cat#P4170

**Critical commercial assays**		

DAPI	Sigma-Aldrich	Cat#D9542
Solution 13 w acridine orange and DAPI	ChemoMetec	Cat#910-3013
QIAquick PCR purification kit	Qiagen	Cat#28106
QIAGEN RNeasy-Plus Mini columns	Qiagen	Cat#74104
Microtubule polymerisation assay	Cytoskeleton, Inc	Cat#BK006P
BCA Protein Assay Kit	Merck	Cat# 71,285
KAPA Library Quantification Kit	Roche	Cat#07960140001
NextSeq 500 High Output Kit (150 cycles)	Illumina	Cat#20024907
NEBNext Ultra II DNA library preparation kit for Illumina	NEB	Cat#E7645S

**Deposited data**		

Raw and analyzed RNA-seqdata	This paper	GEO: GSE178787
Raw and analyzed proteomics data	This paper	ProteomeXchange Consortium PXD0022038
Healthy Donor RNA-seqdata	Corces et al., 2016	GEO: GSE74912

**Experimental models: Cell lines**		

Human: THP1 cells	ATCC	Cat#TIB-202; RRID:CVCL_0006
Human: HL60 cells	ATCC	Cat#CCL-240; RRID:CVCL_0002
Human: KG-1 cells	ATCC	Cat#CCL-246; RRID: CVCL_0374
Human: OCI-AML3 cells	DSMZ	Cat#ACC-582; RRID:CVCL_1844

**Experimental models: Organisms/strains**		

Mouse: NOD.CB17-*Prkdc*^*scid*^/NCrHsd	Axis Bioservices/Envigo	Envigo order code Cat#170
Mouse: NCG (NOD-Prkdc^*em26Cd52*^Il2rg^*em26Cd22*^/NjuCrl)	Charles River Discovery Research Services	RRID:IMSR_CRL:572

**Software and algorithms**		

Attune NxT	Thermo Fisher Scientific UK	https://www.thermofisher.com/uk
PRISM 9	GraphPad	https://www.graphpad.com
MaxQuant v 1.6.6.0	Cox and Mann, 2008	https://maxquant.net/maxquant/
Perseus v 1.6.6.0	Tyanova et al., 2016	https://maxquant.net/perseus/
fastQC v0.11.9	N/A	http://www.bioinformatics.babraham.ac.uk/projects/fastqc/
STAR	Dobin and Gingeras, 2015	https://github.com/alexdobin/STAR/releases
Subread	Liao et al., 2019	http://subread.sourceforge.net/
DESeq2	Love et al., 2014	https://bioconductor.org/packages/release/bioc/html/DESeq2.html

**Other**		

UV Lamp	VWR	Cat#36595-021
Empore™ SDB-XC solid phase extraction discs	CDS	Cat#2240
PVDF membrane	Bio-Rad	Cat#162-0263

### Resource availability

#### Lead contact

Further information and requests for resources and reagents should be directed to and will be fulfilled by the lead contact, Thomas Arthur Milne (thomas.milne@imm.ox.ac.uk).

#### Materials availability

All unique/stable reagents generated in this study are available from the lead contact with a completed Materials Transfer Agreement.

### Experimental model and subject details

#### NOD.CB17-*Prkdc*^*scid*^/NCrHsd (NOD SCID) mice

Female NOD.CB17-*Prkdc*^*scid*^/NCrHsd (NOD SCID) mice aged 5–7 weeks were used for the HL-60 subcutaneous models and weighed an average of 23 gat initiation of treatment approximately 4 weeks later. These mice were initially received by the National Cancer Institute, Frederick, Maryland in 2004 from National Institutes of Health, Bethesda, Maryland. Harlan Laboratories acquired from National Cancer Institute in 2006. Harlan became Envigo in 2015. After the animals arrived at Axis from the supplier, the mice were allowed 7 days to acclimatise following their travel. After this the mice were implanted with the HL-60 cells. The mice were housed in IVC cages (up to 5 mice per cage) and were allowed free access to a standard certified commercial diet and sanitised water throughout the study. Food and water were changed twice a week or as required, and the cage was changed every two weeks. The holding room where all cages were kept was maintained under standard conditions - 18–24°C, humidity at 55–70% and a 12h light/dark cycle was used. The mice were not pre-treated with an agent prior to their implant, then were administered your compounds as reported.

Randomization was performed in the following way. Once the average tumor volume of all animals reached 150 mm3, each animal would be randomly assigned to the treatment group. To do this, all the tumor volumes were arranged from largest to smallest, then assigned to treatment groups in sequence until all animals were assigned to treatment groups. Average tumor volume across each of these groups was measured to verify that the averages between groups were similar. Blinding was performed in the following way. The scientist assigned to measuring the tumors in this study would be blinded from knowing which treatment group the animal was in. Excel spreadsheets were used to take the 3 times weekly tumor volume measurements and the only information the measuring scientist would see is the cage number and the animal’s identification mark. The same scientist would continue to measure these animals until the end of the study. A separate scientist was assigned to dosing the animals – this scientist had a physical ‘dosing sheet’ which had the cage number, animal ID and the treatment details for that particular mouse. All protocols used in this study were approved by the Axis Bioservices Animal Welfare and Ethical Review Committee, and all procedures were carried out under the guidelines of the Animal (Scientific Procedures) Act 1986.

#### NOD-Prkdc^*em26Cd52*^Il2rg^*em26Cd22*^/NjuCrl (NCG) mice

Female NOD-Prkdc^*em26Cd52*^Il2rg^*em26Cd22*^/NjuCrl, Charles River (NCG) recipient mice were used for the HL-60 orthotopic model. Female NCG mice were seven weeksold with body weights (BW) ranging from 19.7 to 23.5 g on Day 1. The animals were fed *ad libitum* water (reverse osmosis, 1 ppm Cl) and NIH 31 Modified and Irradiated Lab Diet® consisting of 18.0% crude protein, 5.0% crude fat, and 5.0% crude fiber. The mice were housed on irradiated Enrich-o’cobs™ Laboratory Animal Bedding in static microisolators on a 12-h light cycle at 21–22°C (70–72°F) and at 40–60% humidity. Control and treatment groups were assigned randomly. For the orthotopic model, the experiments were not blinded. The orthotopic mouse work was conducted at Charles River, Morrisville, a fully PHS assured and AAALAC (Association for Assessment and Accreditation of Laboratory Animal Care International) accredited facility and the protocol was reviewed and approved by the Charles River IACUC committee prior to execution.

#### Cell lines

HL-60 (derived from a 35 yearold female), THP-1 (derived from a 15 yearold male) and KG-1 (derived from a 59 yearold male) AML cell lines were purchased from the AmericanType Culture Collection (ATCC; http://www.atcc.org) cell bank through LGC Standards UK (https://www.lgcstandards.com/GB/en). OCI-AML3 (derived from a 57-year-old male) cells (Wang et al., 1989) were purchased from DSMZ (https://www.dsmz.de/). Cell lines were maintained in RPMI supplemented with 10% FBS and 1% *L*-Glutamine at 37°.

### Method details

#### *In vivo* leukemia analysis: Subcutaneous model

HL-60 Cells (5 × 106) were implanted subcutaneously in a Matrigel matrix (1:1) onto the flank of each mouse and allowed to grow to the pre-specified size of 150 mm3. The mice were not pre-treated with an agent prior to their implant. Mice were grouped randomly into treatment groups based on their bodyweight to ensure even distribution. Mice were treated as indicated in Table 2. Tumors were measured 3 times per week using digital calipers. An independent scientist was assigned to measuring the tumors in the study and was blinded from knowing which treatment group the animal was in. Tumor volume measurements were taken three times weekly and the only information the measuring scientist would see is the cage number and the animal’s identification mark. The length and width of the tumor were measured, and volume calculated using the following formula: volume = (length x width^2^)/2. The tumor control ratio (T/C) was calculated in the following way: ((Mean tumor volume on day 28 – mean starting volume)/(Mean vehicle tumor volume on day 28 – mean vehicle starting volume))∗100. The study was terminated the end of the 28-day treatment period.

#### *In vivo* leukemia analysis: Orthotopic model

HL-60 Cells (1 × 10^7^) were introduced intravenously by tail vein injection. Cells were given 7 days to engraft before commencement of treatment. Animals were then dosed with OXS007417 bid (formulated in 5% DMSO: 0.1% Tween 20 in PBS; PO; 10 mg/kg, with a dosing volume of 10 mL/kg) for 3 weeks. Dosing was initiated seven days after tumor cell inoculation, which was designated as Day 1 of the study. Vehicle and OXS007417 doses were administered via oral gavage (p.o.) twice daily for twenty-one days (bid x 21). Each dose was administered in a volume of 0.2 mL per 20 g body weight (10 mL/kg), and was adjusted to the body weight of the individual animal. Group 1 received vehicle (5% DMSO/0.1% Tween 20 in PBS) p.o. bid x 21 and served as the control for efficacy analysis. Group 2 received OXS007417 at 10 mg/kg p.o. bid x 21. Group 3 received ATRA at 5 mg/kg i.p. qd x 21. Animals were monitored individually for an endpoint of moribundity due to progression of the leukemia. Full hindlimb paralysis, severe ocular proptosis or moribundity was considered sufficient for euthanasia due to tumor progression. Moribund animals were defined as sick animals unable to reach food and water. These deaths were classified as death on survival study (DSS). The time to endpoint (TTE), in days, was recorded for each mouse that died of its disease or was euthanized due to extensive tumor progression. Animals that did not reach the endpoint were euthanized at the end of the study, and were assigned a TTE value equal to the last day. An animal classified as having died from treatment-related (TR) causes was assigned a TTE value equal to the day of death. An animal classified as having died from non-treatment-related (NTR) causes, or used for sampling before endpoint, was excluded from TTE calculations and all further analyses. Survival was analyzed by the Kaplan-Meier method.

#### Compound selection for screening

Small drug-like molecules, selected from a bigger library of 6991 molecules based on structural diversity. These were selected with the “Select Diverse Set” function of Datawarrior (Sander et al., 2015); those 96-well plates having the highest number of compounds with high Diversity Selection Rank scores were selected for testing. See Table S1, compound screening data, for more information.

#### Compound treatment before flow cytometry

Compound stock solutions (10 mM) were prepared in DMSO and stored at −20°C. Serial dilutions were carried out in cell medium prior to use in each experiment and final concentration of DMSO was maintained at 0.1% except for final compound concentrations above 10 μM. Cells were seeded in a 96-well plate at a density of 2 × 10^4^ cells/well, in a 95 μL volume, then 5 μL of compound solutions (x20 of desired concentration) were added. Cells were incubated for 4 days.

#### Flow cytometry

Cells were pelleted by centrifugation at 1000 rpm and suspended in 40 μL of blocking buffer (10% FBS in IMDM, no phenol red), then 10 μL of anti-human CD11b/Mac-1 (555,388, PE labeled, BD Bioscience) solution (25% in blocking buffer) was added. Cells were stored in ice for 20 min. The cell suspension was centrifuged, washed three times with staining buffer (1% FBS in IMDM, no phenol red), and resuspended in 200 μL of staining buffer with 1 mg/mL DAPI (Sigma-Aldrich, D9542) and anti-CD11b-PE antibody. DAPI staining was used to exclude dead cells and evaluate cell death. Flow cytometry was performed on an Attune NxT flow cytometer (Thermo Fisher Scientific UK) with previous compensation. Data was analyzed using Attune NxT software and Flow Jo (v9).

#### Cell counts and viability assessment

Solution 13 containing acridine orange and DAPI was purchased from ChemoMetec (910–3013). After the appropriate cell treatment, one volume of solution 13 was added into 19 volumes of the premixed cell suspension, and analyzed using NucleoCounter® NC-300TM (ChemoMetec).

#### Cytospins and Modified Wright’s staining

Cells were prepared in staining buffer (IMDM, no phenol red +1% FBS) at a concentration of approximately 1 × 10^5^ cells/mL. Cytospins were made (1,000 rpm, 5 min), and the cells allowed to air-dry. Cells were stained with Modified Wright’s stain using a Hematek®. Stained cells were allowed to air-dry and coverslips were affixed with DPX mount prior to microscopy (Sigma-Aldrich, 06522).

#### Isolation of RNA

Total RNA for the RNA-seq was isolated using QIAGEN RNeasy-Plus Mini columns as per the manufacturer’s instructions. RNA purity was analyzed using RNA Screen Tape with a TapeStation system (Agilent).

#### RNA-seq

Poly-A containing mRNA molecules were purified from total RNA using oligo-dT attached magnetic beads. Following purification, the mRNA was fragmented using divalent cations under elevated temperature. First strand cDNA was synthesised using random primers (NEB). Following second strand cDNA synthesis, cDNA libraries underwent end repair, a single adenylation of the 3′ ends and TRUE-seq adapter ligation. Libraries were enriched by PCR (15 cycles). Library quality was assessed by DNA Screen Tape with Tape Station system (Agilent), quantified by Qubit assay (Thermo Fisher Scientific) and pooled. Next-generation sequencing of pooled libraries was performed (Illumina NextSeq), resulting in approximately 10 million pairs of 75-bp reads per sample.

#### Analysis of cell cycle by flow cytometry

Cells were harvested at indicated time points, washed in PBS and suspended in hypotonic fluorochrome solution [50 μg/mL propidium iodide (PI), 0.1% (w/v) sodium citrate, 0.1% (v/v) Triton X-100] and stored for at least 1 h in the dark at 4°C. Cells were washed in PBS and samples then incubated with anti-PH3 (1:40) in FACS staining buffer (IMDM, no phenol red +10% FBS) for 20minat 4°C in the dark. Cells were washed in FACS buffer (IMDM, no phenol red +1% FBS) and flow cytometry was performed using an Attune NxT. Results were analyzed using FlowJo_V10 software.

#### Immunohistochemistry

Cells were washed with PBS and resuspended in 100% FCS and cytospun onto coated slides using a Shandon Cytospin 4 (Thermo Scientific) at 30g for 5 min. Cytospins were fixed in methanol for 7 min at −20°C and dipped 10 times in ice-cold acetone. Slides were then washed three times in TBS 0.01% Tween 20 (TBST) for 5 min on a mechanical rocker. Cells were subsequently blocked for 15 min at room temperature in TBS, 0.05% Tween 20, 1% bovine serum albumin (BSA). Samples were covered with 50 μL of TBS, 0.025% Tween 20, 1% BSA containing anti-tubulin, overlain with a coverslip and incubated overnight at 4°C in a humid chamber. Cover slips were then removed and slides were washed three times for 5 min with TBST, covered with 50 μL containing the appropriate secondary antibody and incubated in the dark for 40 min. Slides were then washed three times for 5 min with TBST, and once for 2 min with PBS. Samples were then counter stained with 0.25 μg/mL DAPI for 1 min, mounted using Pro-Long Gold antifade reagent (Invitrogen) and imaged using a widefield fluorescence microscope (DeltaVision Elite, imsol).

#### General protocol for treatment and lysis

HL-60 cells (2 × 10^6^ cells/mL in serum free RPMI media) were treated for 1 h with the probe **4** or DMSO vehicle at 37°C. In the case of competition experiments, cells were pre-treated with competitor or DMSO vehicle for 30 min followed by 1 h treatment with probe **4**. Treated cells were pelleted and washed with PBS. The resulting pellets were resuspended in PBS and irradiated at 365 nm for 5 min (100 W lamp, VWR 36595-021) on ice. Cells were lysed in buffer containing 0.1% SDS, 1% Triton –X-100 and 1× EDTA-free protease inhibitor cocktail (Calbiochem set III, 539134) in PBS. Protein concentration of each lysate was determined using a BCA assay (Merck, 71285).

#### In-gel fluorescence

40 μL of each lysate (concentrations adjusted to 1 μg/μL) was treated with 2.4 μL of premixed click chemistry mixture (final concentrations of 100 μM TAMRA-N_3_ (Sigma-Aldrich, 760757), 1 mM CuSO_4_, 1 mM TCEP and 100 μM TBTA) for 1 h. Proteins were precipitated using MeOH/CHCl_3_ and the resulting pellets washed twice with MeOH. The air-dried pellets were dissolved in 20 μL of 1× NuPAGE LDS buffer with 0.1% mercaptoethanol and heated at 95°C for 5 min. The proteins were separated by NuPAGE 4–12% Bis-Tris gel in MES SDS running buffer. The gel was imaged using a Typhoon FLA 9500 scanner and then stained with Coomassie (InstantBlue™, Expedeon) and imaged using a BioRad ChemiDoc scanner.

#### Proteomics

HL-60 cells were treated in triplicate and lysed as described above. 400 μL of each lysate (concentrations adjusted to 2.5 μg/μL) was treated with 24 μL of a click chemistry master mix (final concentrations of 100 μM AzRB, 1 mM CuSO_4_, 1 mM TCEP and 100 μM TBTA) for 1 h. The click reaction was quenched by adding 8 μL of 500 mM EDTA (10 mM final concentration). Proteins were precipitated using MeOH/CHCl_3_/water and the resulting pellets washed twice with MeOH. The air-dried pellets were dissolved in 80 μL of 1% SDS in 50 mM HEPES pH 8.0 by vortexing and sonicating and then diluted to 400 μL with 50 mM HEPES pH 8.0 (0.2% SDS final concentration).

Samples were incubated with 100 μL (1:10 ratio of bead suspension:protein) of NeutrAvidin agarose resin (Thermo Scientific 29201, pre-washed three times with 1 mL of 0.2% SDS in 50 mM HEPES pH 8.0) for 2hat room temperature. The supernatants were removed and the beads washed three times with 1 mL of 0.2% SDS in 50 mM HEPES pH 8.0 and then twice with 50 mM HEPES pH 8.0. The beads were then resuspended in 150 μL of 50 mM HEPES pH 8.0 and on-bead proteins were reduced with TCEP (5 mM final concentration) and alkylated with CAA (15 mM final concentration) for 10 min with gentle shaking. Proteins were digested overnight at 37°C with 5 μL of trypsin (1 μg dissolved in 50 mM HEPES pH 8.0, Promega V5111). The trypsin digestion was quenched by adding 4 μL of 1× EDTA-free protease inhibitor cocktail (Roche 11873580001). The supernatants were collected and the beads washed (50μL) with 50 mM HEPES pH 8.0. The second wash was combined with the corresponding supernatant and vacuum-dried. The peptide solutions were desalted on stage-tips according to a published protocol (Rappsilber et al., 2003). The peptides were eluted from the sorbent (Empore™ SDB-XC solid phase extraction discs, 3M, 2240) with 60% acetonitrile in water (60 μL), dried in a Savant SPD1010 SpeedVac® Concentrator (Thermo Scientific) and stored at −80°C until LC-MS/MS analysis. Peptides were reconstituted in 2% acetonitrile in water with 0.5% trifluoroacetic acid for LC-MS/MS analysis.

#### NanoLC-MS/MS analysis

Peptides were separated on an EASY-Spray™ Acclaim PepMap C18 column (50 cm × 75 μm inner diameter, Thermo Fisher Scientific) using a binary solvent system of 2% acetonitrile with 0.1% formic acid (Solvent A) and 80% acetonitrile with 0.1% formic acid (Solvent B) in an Easy nLC-1000 system (Thermo Fisher Scientific). 2 μL of peptide solution was loaded using Solvent A onto an Acclaim PepMap100C18 trap column (2 cm × 75 μm inner diameter), followed by a linear gradient separation of 0-100% Solvent B over 70minat a flow rate of 250 nL/min. Liquid chromatography was coupled to a QExactive mass spectrometer via an easy-spray source (Thermo Fisher Scientific). The QExactive was operated in data-dependent mode with survey scans acquired at a resolution of 70,000at*m/z* 200 (transient time 256 ms). Up to 10 of the most abundant isotope patterns with charge +2 to +7 from the survey scan were selected with an isolation window of 2.0 *m*/*z* and fragmented by HCD with normalized collision energies of 25. The maximum ion injection times for the survey scan and the MS/MS scans (acquired with a resolution of 17,500at*m*/*z* 200) were 20 and 120 ms, respectively. The ion target value for MS was set to 10^6^ and for MS/MS to 10^5^, and the intensity threshold was set to 8.3 × 10^2^.

#### Western blotting

HL-60 cells were treated and lysed as described above. 100 μL of each lysate (concentrations adjusted to 2.5 μg/μL) was treated with 6 μL of premixed click chemistry mixture (final concentrations of 100 μM biotin-N_3_ (Sigma-Aldrich, 762024), 1 mM CuSO_4_, 1 mM TCEP and 100 μM TBTA) for 1 h. The click reactions were quenched by adding 2 μL of 500 mM EDTA (10 mM final concentration). Proteins were precipitated using MeOH/CHCl_3_/water and the resulting pellets were washed twice with MeOH. The air-dried pellets were dissolved in 80 μL of 1% SDS in PBS by vortexing and sonicating and then diluted to 400 μL with PBS.

Samples were incubated with 15 μL of MyOne™ Streptavidin T1 Dynabeads™ (Thermo Scientific, 65601, pre-washed three times with 0.2% SDS in PBS) for 1 h in the shaker. The supernatants were removed and the beads washed three times with 0.1% SDS, 1% Triton X-100 in PBS and then three times with 0.2% SDS in PBS. The beads were resuspended in 50 μL of 1× NuPAGE LDS buffer with 0.1% mercaptoethanol and heated at 95°C for 5 min. The eluted proteins were separated by NuPAGE 4–12% Bis-Tris gel in MES SDS running buffer and transferred to a PVDF membrane (Bio-Rad, 162–0263). Tubulin beta chain protein was detected with an anti-TUBB antibody (1:500 in 5% fat-free milk solution in TBST, Invitrogen MA5-16308) followed by an anti-mouse secondary antibody (Alexa Fluor™ Plus 800, 1:10000, ThermoFisher A32730). The blots were imaged with a Licor Odyssey system.

#### Microtubule polymerisation assay

The microtubule polymerisation assay was performed using porcine neuronal tubulin (Cytoskeleton, Inc, BK006P) as an adaptation of the original method of Shelanski et al. and Lee et al. (Lee and Timasheff, 1977; Shelanski et al., 1973) at Cytoskeleton, Inc.

#### General information for chemical synthesis

All reactions involving moisture sensitive reagents were carried out under a nitrogen or argon atmosphere. Solvents were dried following the procedure outlined by Grubbs et al.^54^ Water was purified by an Elix® UV-10 system. All other solvents and reagents were used as supplied (analytical or HPLC grade) without prior purification. Organic layers were dried over anhydrous Na_2_SO_4_ or MgSO_4_. Brine refers to a saturated aqueous solution of NaCl. *In vacuo* refers to the use of a rotary evaporator attached to a diaphragm pump. Analytical thin-layer chromatography (TLC) was performed on Merck aluminum plates coated with 60 F_254_ silica. Plates were visualised using UV irradiation (λ 254 nm) and staining with a KMnO_4_ solution. Flash column chromatography was performed on Kieselgel 60 silica gel (230–400 mesh particle size) in a glass column, or using a Biotage Isolera One 3.0 or SP4 automated purification system with the default settings for a KP-Sil cartridge, monitoring at 254 and 280 nm. NMR spectra were recorded on Bruker Advance spectrometers in the deuterated solvent stated. The field was locked by external referencing to the relevant deuteron resonance. Chemical shifts (*δ*) are reported in parts per million (ppm) and coupling constants (*J*), determined by analysis using MestreNova software, are quoted in Hz. Data are reported as follows: chemical shift, multiplicity (s = singlet, bs = broad singlet, d = doublet, t = triplet, q = quartet and m = multiplet), coupling constant and integration. Low-resolution mass spectra (*m/z*) were recorded on an Agilent 1260 Infinity II with Diode Array and Single Quadrupole Detectors in solutions of MeOH. A selected peak is reported in Daltons and its intensity given as percentage of the base peak. High resolution mass spectra (HRMS) were run on a Bruker microTOF (ESI and APCI) or on a Waters GCT (EI), by the mass spectrometry department of the Chemistry Research Laboratory, University of Oxford, UK. Temperatures below 25°C were obtained using the following cooling baths: 0°C ice/water, −15°C dry ice/ethylene glycol and −78°C dry ice/acetone.

#### Synthesis of OXS000275 (1)

**Figure:**
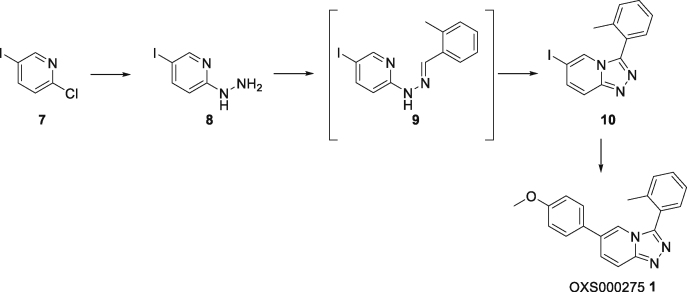

#### (5-Iodo-2-pyridyl)hydrazine (8)

To a solution of 2-chloro-5-iodopyridine **7** (2.48 g, 10.4 mmol) in pyridine (30 mL) was added hydrazine hydrate (5.04 mL, 104 mmol) dropwise. The mixture was refluxed overnight. The solution was concentrated to give a solid, which was triturated in pentane (5 mL) to give hydrazine **8** (2.4 g, 10 mmol, quant.) as a gray solid. ^1^H NMR (400 MHz, CD_3_OD) *δ* 8.02 (s, 1H), 7.56 (dd, *J* = 8.9, 2.2 Hz, 1H), 6.52 (d, *J* = 8.9 Hz, 1H); *m/z* (ESI^+^) 235.9 ([M + H]^+^, 100%); HRMS (ESI^+^) C_5_H_7_N_3_I^+^ ([M + H]^+^) requires 235.9679; found 235.9679. The data was in agreement with the reported values (Hapuarachchige et al., 2011).

#### 6-Iodo-3-(*o*-tolyl)-[1,2,4]triazolo[4,3-*a*]pyridine (10)

To a suspension of hydrazine **8** (875 mg, 3.72 mmol) in EtOH (12 mL) was added 2-methylbenzaldehyde (447 mg, 3.72 mmol). The mixture was heated to reflux for 2 h, then cooled to room temperature. The resulting solid was filtered and washed with EtOH to give the hydrazone **9**. To a suspension of the crude hydrazone **9** in anhydrous CH_2_Cl_2_/MeOH (10:2, 19 mL) was added (diacetoxyiodo)benzene (1.68 g, 5.21 mmol) at 0°C. The mixture was stirred at room temperature overnight, then diluted with water, extracted with CH_2_Cl_2_ and dried over anhydrous Na_2_SO_4_. The crude product was purified using flash column chromatography to give the triazolopyridine **10** (748 mg, 2.23 mmol, 60%) as an off-white solid. ^1^H NMR (500 MHz, CDCl_3_) *δ* 8.03 (t, *J* = 1.3 Hz, 1H), 7.61 (dd, *J* = 9.6, 1.0 Hz, 1H), 7.47 (td, *J* = 7.5, 1.5 Hz, 1H), 7.44–7.39 (m, 3H), 7.37 (t, *J* = 7.4 Hz, 1H), 2.25 (s, 3H); ^13^C NMR (125 MHz, CDCl_3_) *δ* 148.5, 145.8, 138.7, 135.1, 131.3, 130.8, 130.2, 127.6, 126.4, 125.0, 117.5, 77.7, 19.9; *m/z* (ESI^+^) 336.0 ([M + H]^+^, 100%); HRMS (ESI^+^) C_13_H_11_N_3_I^+^ ([M + H]^+^) requires 335.9992; found 335.9988.

#### 6-(4-Methoxyphenyl)-3-(*o*-tolyl)-[1,2,4]triazolo[4,3-*a*]pyridine (OXS000275, 1)

To a solution of iodide **10** (126 mg, 0.378 mmol) in degassed 1,4-dioxane/water (5:2, 3.8 mL) were added (4-methoxyphenyl)boronic acid (69 mg, 0.45 mmol), potassium carbonate (78 mg, 0.57 mmol) and Pd(dppf)Cl_2_ (14 mg, 0.19 mmol), and the mixture was heated to 100°C. After 16 h, the mixture was diluted with EtOAc, filtered through Celite and concentrated. The crude product was purified using flash column chromatography to give OXS000275**1** (112 mg, 0.355 mmol, 94%) as a brown solid. ^1^H NMR (400 MHz, CDCl_3_) *δ* 7.85 (dd, *J* = 9.5, 0.9 Hz, 1H), 7.82 (s, 1H), 7.52 (dd, *J* = 9.5, 1.6 Hz, 1H), 7.50–7.33 (m, 6H), 6.96 (d, *J* = 8.8 Hz, 2H), 3.82 (s, 3H), 2.29 (s, 3H); ^13^C NMR (100 MHz, CDCl_3_) *δ* 160.2, 149.4, 146.7, 138.8, 131.3, 130.7, 130.5, 128.7, 128.5, 128.4, 128.3, 126.5, 125.9, 118.7, 116.5, 114.8, 55.6, 20.0; *m/z* (ESI^+^) 316.1 ([M + H]^+^, 100%); HRMS (ESI^+^) C_20_H_18_N_3_O^+^ ([M + H]^+^) requires 316.1444; found 316.1442.

#### Synthesis of OXS007417(2)

**Figure:**
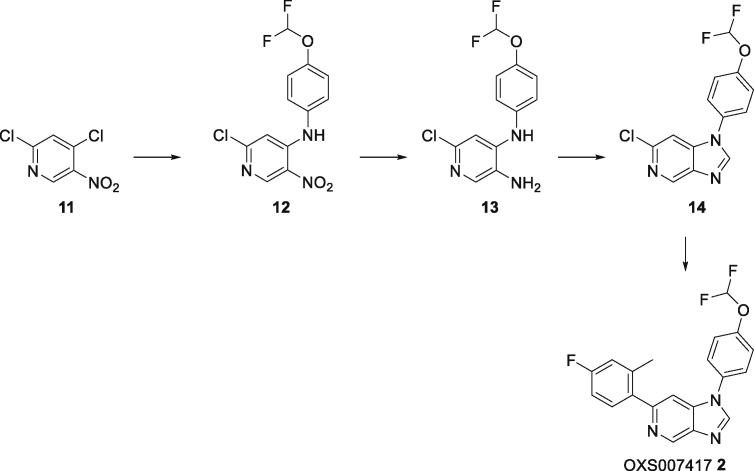

#### 2-Chloro-*N*-(4-(difluoromethoxy)phenyl)-5-nitropyridin-4-amine (12)

To a solution of 2,4-dichloro-5-nitro-pyridine **11** (10.0 g, 51.8 mmol) in MeCN (150 mL) was added 4-(difluoromethoxy)aniline (6.29 mL, 51.8 mmol), followed by triethylamine (14.4 mL, 104 mmol). The mixture was stirred at room temperature for 2 days, then the mixture was evaporated to dryness. The residue was dissolved in EtOAc and washed with water, followed by brine, then dried overMgSO_4_ and concentrated *in vacuo* to give **12** (14.9 g, 47.1 mmol, 91%) as a gold solid. ^1^H NMR (400 MHz, CDCl_3_) *δ* 9.58 (bs, 1H), 9.10 (s, 1H), 7.32–7.26 (m, 4H), 6.85 (s, 1H), 6.57 (t, *J* = 73.2 Hz, 1H); ^13^C NMR (100 MHz, CDCl_3_) *δ* 156.9, 150.3 (t, *J* = 3 Hz), 149.5, 149.0, 133.2, 129.8, 127.4, 121.7, 115.6 (t, *J* = 262 Hz), 108.1; *m/z* (ESI^+^) 316.0 ([M + H]^+^, 100%).

#### 6-Chloro-*N*^4^-(4-(difluoromethoxy)phenyl)pyridine-3,4-diamine (13)

Nitro **12** (14.9 g, 47.1 mmol) was dissolved in IMS (125 mL) and water (250 mL), and ammonium chloride (10.1 g, 189 mmol) and iron (13.2 g, 236 mmol) were added and the mixture heated to 80°C overnight. The mixture was cooled, diluted with CH_2_Cl_2_ and filtered through Celite. The organic phase was washed with water then brine, dried over anhydrous MgSO_4_ and concentrated *in vacuo* to give amine **13** (14.7 g, 46.4 mmol, 98%) as a red solid, which was used in the following step without further purification. ^1^H NMR (400 MHz, CDCl_3_) *δ* 7.79 (s, 1H), 7.17–7.11 (m, 4H), 6.86 (s, 1H), 6.51 (t, *J* = 74 Hz, 1H), 6.06 (bs, 1H), 3.29 (bs, 2H); ^13^C NMR (100 MHz, CDCl_3_) *δ* 147.3 (t, *J* = 3 Hz), 144.2, 144.0, 138.0, 137.0, 129.6, 123.0, 121.3, 115.9 (t, *J* = 261 Hz), 107.0; *m/z* (ESI^+^) 286.0 ([M + H]^+^, 100%).

#### 6-Chloro-1-(4-(difluoromethoxy)phenyl)-1*H*-imidazo[4,5-*c*]pyridine (14)

To a suspension of diamine **13** (10.6 g, 37.1 mmol) in diethoxymethoxyethane (110 g, 742 mmol), was added formic acid (1.71 g, 37.1 mmol) and the mixture heated to 100°C overnight. The mixture was evaporated to dryness, saturated aqueous NaHCO_3_ was added, and the resulting precipitate was filtered, washed with water, followed by 1:1 Et_2_O/hexane, and concentrated *in vacuo* to give imidazopyridine **14** (8.90 g, 30.1 mmol, 81%) as a light brown solid. ^1^H NMR (400 MHz, CDCl_3_) *δ* 8.94 (s, 1H), 8.13 (s, 1H), 7.51–7.39 (m, 5H), 6.63 (t, *J* = 72.8 Hz, 1H); ^13^C NMR (100 MHz, CDCl_3_) *δ* 151.2 (t, *J* = 3 Hz), 145.1, 144.7, 142.8, 140.8, 140.7, 132.1, 125.8, 121.9, 115.5 (t, *J* = 263 Hz), 105.6; *m/z* (ESI^+^) 296.0 ([M + H]^+^, 100%).

#### 1-(4-(Difluoromethoxy)phenyl)-6-(4-fluoro-2-methylphenyl)-1*H*-imidazo[4,5-*c*]pyridine (OXS007417, 2)

Chloride **14** (6.50 g, 22.0 mmol), (4-fluoro-2-methyl-phenyl)boronic acid (4.06 g, 26.4 mmol) and potassium carbonate (6.08 g, 44.0 mmol) were dissolved in DME (50 mL) and water (25 mL). Nitrogen was bubbled though the solution for 10 min, then Pd(dppf)Cl_2_ (0.804 g, 1.10 mmol) was added and nitrogen bubbled for a further 5 min. The mixture was heated to 80°C overnight. The mixture was cooled, diluted with EtOAc and filtered through Celite. The aqueous phase was extracted with EtOAc and the combined organics were dried over anhydrous MgSO_4_ and concentrated *in vacuo*. The crude residue was purified using flash column chromatography (0%–60% EtOAc in hexane), and the resulting solid triturated with hexane and dried to give OXS007417**2** (3.10 g, 8.39 mmol, 38%) as an off-white solid. ^1^H NMR (500 MHz, CDCl_3_) *δ* 9.25 (d, *J* = 1.1 Hz, 1H), 8.17 (s, 1H), 7.55–7.50 (m, 2H), 7.46 (d, *J* = 1.1 Hz, 1H), 7.37 (dd, *J* = 8.5, 5.5 Hz, 3H), 7.04–6.90 (m, 2H), 6.60 (t, *J* = 72.8 Hz, 1H), 2.35 (s, 3H); ^13^C NMR (126 MHz, CDCl_3_) *δ* 162.6 (d, *J* = 247 Hz), 153.6, 150.9 (t, *J* = 3 Hz), 143.9, 142.9, 139.9, 139.3, 138.8 (d, *J* = 8 Hz), 137.0 (d, *J* = 3 Hz), 132.6, 131.6 (d, *J* = 8 Hz), 125.7, 121.9, 117.4 (d, *J* = 21 Hz), 115.5 (t, *J* = 263 Hz), 112.8 (d, *J* = 21 Hz), 105.7, 20.7; *m/z* (ESI^+^) 370.1 ([M + H]^+^, 100%); HRMS (APCI) C_20_H_15_ON_3_F_3_ ([M + H]^+^) requires 370.1158; found 370.1162.

#### Synthesis of OXS007464 (3) and OXS007564(6)

**Figure:**
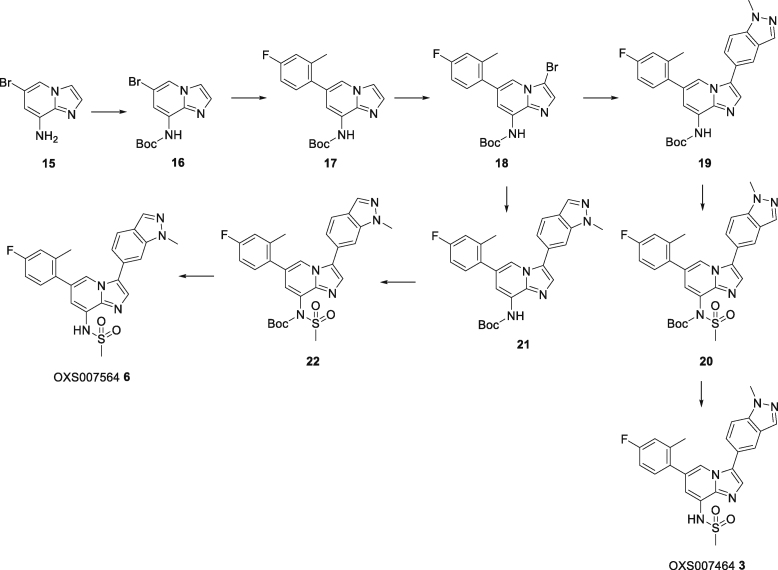

#### *tert*-Butyl (6-bromoimidazo[1,2-*a*]pyridin-8-yl)carbamate (16)

To a solution of 6-bromoimidazo[1,2-*a*]pyridin-8-amine **15** (2.05 g, 9.67 mmol) in anhydrous THF (20 mL), sodium bis(trimethylsilyl)amide solution (1 M in THF, 29 mL, 29 mmol) was added dropwise. The solution was stirred at room temperature for 30 min, then Boc anhydride (2.11 g, 9.67 mmol) was added portionwise. The reaction mixture was stirred at room temperature for another 2 h, then quenched with saturated aqueous NH_4_Cl, extracted with three times with CH_2_Cl_2_, dried over anhydrous Na_2_SO_4_ and concentrated. The crude product was purified using flash column chromatography (2%–25% EtOAc in pentane) to give Boc-protected **16** (2.03 g, 6.50 mmol, 67%) as a green solid. ^1^H NMR (400 MHz, CDCl_3_) *δ* 7.93–7.89 (m, 2H), 7.90 (s, 1H), 7.49 (d, *J* = 1.2 Hz, 1H), 7.48 (d, *J* = 1.2 Hz, 1H), 1.52 (s, 9H); ^13^C NMR (100 MHz, CDCl_3_) *δ* 152.3, 137.8, 132.5, 128.3, 119.2, 113.8, 111.4, 108.2, 81.7, 28.3; *m/z* (ESI^+^) 312.0 ([M + H]^+^, 100%); HRMS (ESI^+^) C_12_H_15_N_3_O_2_Br^+^ ([M + H]^+^) requires 312.0342; found 312.0343.

#### *tert*-Butyl (6-(4-fluoro-2-methylphenyl)imidazo[1,2-*a*]pyridin-8-yl)carbamate (17)

Pd(PPh_3_)_4_ (259 mg, 0.22 mmol) was added to a stirred solution of **16** (700 mg, 2.24 mmol), (4-fluoro-2-methyl-phenyl)boronic acid (345 mg, 2.24 mmol) and potassium phosphate tribasic (1.43 g, 6.73 mmol) in degassed DME/water (3:1, 14 mL) at room temperature. The resultant mixture was heated to 90°C for 16 h, then cooled to room temperature, diluted with EtOAc, filtered through Celite and concentrated. Purification using flash column chromatography (20% EtOAc in pentane) gave the aryl-substituted product **17** (741 mg, 2.17 mmol, 97%) as a pale yellow foam. ^1^H NMR (400 MHz, CDCl_3_) *δ* 7.91 (s, 1H), 7.76 (s, 1H), 7.69 (d, *J* = 1.5 Hz, 1H), 7.58 (d, *J* = 1.2 Hz, 1H), 7.56 (d, *J* = 1.3 Hz, 1H), 7.21 (dd, *J* = 8.4, 5.9 Hz, 1H), 6.98 (dd, *J* = 9.7, 2.7 Hz, 1H), 6.92 (td, *J* = 8.4, 2.7 Hz, 1H), 2.30 (s, 3H), 1.53 (s, 9H); ^13^C NMR (101 MHz, CDCl_3_) *δ* 162.3 (d, *J* = 246 Hz), 152.5, 138.6 (d, *J* = 8 Hz), 138.3, 133.8 (d, *J* = 3 Hz), 132.3, 131.4 (d, *J* = 8 Hz), 127.2, 127.1, 118.0, 116.9 (d, *J* = 21 Hz), 113.8, 112.6 (d, *J* = 21 Hz), 110.6, 81.1, 28.2, 20.6; HRMS (ESI^+^) C_19_H_21_FN_3_O_2_^+^ ([M + H]^+^) requires 342.1612; found 342.1612.

#### *tert*-Butyl (3-bromo-6-(4-fluoro-2-methylphenyl)imidazo[1,2-*a*]pyridin-8-yl)carbamate (18)

N-bromosuccinimide (334 mg, 1.87 mmol) was added to a stirred solution of **17** (640 mg, 1.87 mmol) in THF (4 mL) at room temperature. The resultant mixture was stirred for 2 h, then concentrated. Purification using flash column chromatography (3% EtOAc in pentane) gave the brominated product **18** (619 mg, 1.47 mmol, 79%) as a pale yellow foam. ^1^H NMR (400 MHz, CDCl_3_) *δ* 7.90–7.82 (m, 2H), 7.69 (d, *J* = 1.5 Hz, 1H), 7.55 (s, 1H), 7.24 (dd, *J* = 8.4, 5.9 Hz, 1H), 7.00 (dd, *J* = 9.7, 2.7 Hz, 1H), 6.98–6.91 (m, 1H), 2.31 (s, 3H), 1.53 (s, 9H); ^13^C NMR (101 MHz, CDCl_3_) *δ* 162.5 (d, *J* = 247 Hz), 152.4, 138.7 (d, *J* = 8 Hz), 138.5, 133.7 (d, *J* = 3 Hz), 132.4, 131.5 (d, *J* = 8 Hz), 128.0, 127.3, 117.0 (d, *J* = 21 Hz), 115.9, 112.7 (d, *J* = 21 Hz), 110.9, 96.3, 81.4, 28.2, 20.6 (d, *J* = 2 Hz); HRMS (ESI^+^) C_19_H_20_BrFN_3_O_2_^+^ ([M + H]^+^) requires 420.0717; found 420.0720.

#### *tert*-Butyl (6-(4-fluoro-2-methylphenyl)-3-(1-methyl-1*H*-indazol-5-yl)imidazo[1,2-*a*]pyridin-8-yl)carbamate (19)

Pd(dppf)Cl_2_ (71 mg, 0.10 mmol) was added to a stirred solution of **18** (580 mg, 1.38 mmol), (1-methylindazol-5-yl)boronic acid (291 mg, 1.66 mmol) and potassium carbonate (572 mg, 4.14 mmol) in degassed DME/water (3:1, 9 mL) at room temperature. The resultant mixture was heated to 70°C for 16 h, then cooled to room temperature, diluted with EtOAc, filtered through Celite and concentrated. Purification using flash column chromatography (30% EtOAc in pentane) gave the aryl-substituted product **19** (641 mg, 1.36 mmol, 99%) as a pale yellow foam. ^1^H NMR (400 MHz, CDCl_3_) *δ* 8.05 (d, *J* = 0.8 Hz, 1H), 7.99 (s, 1H), 7.91 (t, *J* = 1.2 Hz, 1H), 7.85–7.80 (m, 2H), 7.63 (s, 1H), 7.57 (dd, *J* = 8.7, 1.5 Hz, 1H), 7.53 (dt, *J* = 8.7, 0.9 Hz, 1H), 7.20 (dd, *J* = 8.4, 5.9 Hz, 1H), 6.96 (dd, *J* = 9.7, 2.7 Hz, 1H), 6.89 (td, *J* = 8.4, 2.7 Hz, 1H), 4.13 (s, 3H), 2.31 (s, 3H), 1.55 (s, 9H); ^13^C NMR (101 MHz, CDCl_3_) *δ* 162.3 (d, *J* = 246 Hz), 152.6, 139.5, 138.6 (d, *J* = 5 Hz), 138.5, 134.1 (d, *J* = 3 Hz), 133.1, 131.5, 131.4, 131.0, 127.5, 127.4 (d, *J* = 14 Hz), 126.9, 124.4, 121.3, 121.1, 116.9 (d, *J* = 21 Hz), 115.6, 112.6 (d, *J* = 21 Hz), 110.5, 109.9, 81.1, 35.7, 28.2, 20.7 (d, *J* = 2 Hz); HRMS (ESI^+^) C_27_H_27_FN_5_O_2_^+^ ([M + H]^+^) requires 472.2143; found 472.2135.

#### *tert*-Butyl (6-(4-fluoro-2-methylphenyl)-3-(1-methyl-1*H*-indazol-5-yl)imidazo[1,2-*a*]pyridin-8-yl)(methylsulfonyl)carbamate (20)

Sodium hydride (60% w/w dispersion in mineral oil, 407 mg, 10.2 mmol) was added to a stirred solution of **19** (1.60 g, 3.39 mmol) in THF (150 mL) at room temperature. The resultant mixture was stirred for 30 min before the addition of methanesulfonyl chloride (466 mg, 4.07 mmol). The resultant mixture was stirred at room temperature for 16 h, before the addition of water. The aqueous layer was extracted with EtOAc, dried over anhydrous MgSO_4_ and concentrated. Purification using flash column chromatography (0%–60% EtOAc in pentane) gave mesyl **20** (1.70 g, 3.09 mmol, 91%) as an orange oil. ^1^H NMR (400 MHz, CDCl_3_) *δ* 8.15 (d, *J* = 1.2 Hz, 1H), 8.06 (s, 1H), 7.90 (m, 1H), 7.70 (s, 1H), 7.55 (d, *J* = 0.8H z, 2H), 7.30 (d, *J* = 1.6 Hz, 1H), 7.22 (dd, *J* = 5.6, 8.4H, 1H), 6.99 (dd, *J* = 2.4, 9.6 Hz, 1H), 6.92 (td, *J* = 2.8, 8.4 Hz, 1H), 4.13 (s, 3H), 3.79 (s, 3H), 2.30 (s, 3H), 1.51 (s, 9H); *m/z* (ESI^+^) 550.1 ([M + H]^+^, 100%).

#### *N*-(6-(4-fluoro-2-methylphenyl)-3-(1-methyl-1*H*-indazol-5-yl)imidazo[1,2-*a*]pyridin-8-yl)methanesulfonamide (OXS007464, 3)

A solution of **20** (130 mg, 0.25 mmol) in 2 M HCl in Et_2_O (4.0 mL) was stirred at room temperature for 16 h, before concentration *in vacuo*. The residue was dissolved in EtOAc, and basified with saturated aqueous NaHCO_3_. The aqueous layer was extracted with EtOAc, dried over anhydrous MgSO_4_ and concentrated. Purification using flash column chromatography (0%–100% EtOAc in pentane) gave the product OXS007464**3** (54 mg, 0.12 mmol, 50%) as an off-white solid. ^1^H NMR (400 MHz, DMSO-*d*_6_) *δ* 8.12 (s, 1H), 8.09 (s, 1H), 8.07 (s, 1H), 7.81 (s, 1H), 7.80 (d, *J* = 8.8 Hz, 1H), 7.69 (dd, *J* = 8.7, 1.3 Hz, 1H), 7.38 (dd, *J* = 8.4, 6.1 Hz, 1H), 7.18 (dd, *J* = 10.1, 2.6 Hz, 1H), 7.14 (d, *J* = 1.4 Hz, 1H), 7.08 (td, *J* = 8.5, 2.7 Hz, 1H), 4.09 (s, 3H), 3.28 (s, 3H), 2.29 (s, 3H); ^13^C NMR (101 MHz, DMSO-*d*_6_) *δ* 161.7 (d, *J* = 244 Hz), 139.3, 139.1, 138.6 (d, *J* = 8 Hz), 133.6 (d, *J* = 3 Hz), 132.8, 131.6 (d, *J* = 8 Hz), 131.5, 127.3, 126.6, 126.5, 125.4, 123.8, 120.5, 120.4, 118.1, 116.8 (d, *J* = 21 Hz), 115.1, 112.6 (d, *J* = 21 Hz), 110.6, 40.9, 35.4, 20.0; *m/z* (ESI^+^) 450.1 ([M + H]^+^, 100%); HRMS (ESI^+^) C_23_H_21_O_2_N_5_FS^+^ ([M + H]^+^) requires 450.1395, found 450.1390.

#### *tert*-Butyl (6-(4-fluoro-2-methylphenyl)-3-(1-methyl-1*H*-indazol-6-yl)imidazo[1,2-*a*]pyridin-8-yl)carbamate (21)

Bromide **18** (250 mg, 0.59 mmol), (1-methylindazol-6-yl)boronic acid (126 mg, 0.716 mmol) and potassium phosphate tribasic (379 mg, 1.79 mmol) were dissolved in degassed DME/water (4:1, 5 mL). Pd(dppf)Cl_2_ (22 mg, 0.030 mmol) was added and the reaction heated at 80°C overnight. The mixture was cooled to room temperature and concentrated *in vacuo*. The residue was purified using flash column chromatography (0%–40% EtOAc in hexane) to give aryl-substituted **21** (190 mg, 0.403 mmol, 68%) as an orange solid. ^1^H NMR (400 MHz, CDCl_3_) *δ* 8.03 (s, 1H), 7.96 (bs, 1H), 7.91 (d, *J* = 1.6 Hz, 1H), 7.85 (m, 2H), 7.69 (s, 1H), 7.56 (s, 1H), 7.34 (dd, *J* = 1.2, 8.4 Hz, 1H), 7.21 (dd, *J* = 5.6, 8.4 Hz, 1H), 6.95–6.98 (m, 1H), 6.92-6.87 (m, 1H), 4.12 (s, 3H), 2.32 (s, 3H), 1.55 (s, 9H); *m/z* (ESI^+^) 472.2 ([M + H]^+^, 100%).

#### *tert*-Butyl (6-(4-fluoro-2-methylphenyl)-3-(1-methyl-1*H*-indazol-6-yl)imidazo[1,2-*a*]pyridin-8-yl)(methylsulfonyl)carbamate (22)

To **21** (182 mg, 0.382 mmol) in THF (4 mL) was added sodium hydride (60% w/w dispersion in mineral oil, 44 mg, 1.2 mmol) and stirred for 15 min. Methanesulfonyl chloride (53 mg, 0.46 mmol) was added and the reaction stirred overnight. Two further equivalents of sodium hydride and methanesulfonyl chloride were added to complete the reaction. The reaction mixture was quenched with ice water, extracted with CH_2_Cl_2_, dried over anhydrous MgSO_4_ and concentrated *in vacuo*. The crude product was purified using flash column chromatography (30%–80% EtOAc in hexane) to give the mesyl product **22** (130 mg, 0.237 mmol, 61%) as an orange solid. ^1^H NMR (400 MHz, CDCl_3_) *δ* 8.23 (d, *J* = 1.6 Hz, 1H), 8.04 (d, *J* = 1.2 Hz,1H), 7.86 (dd, *J* = 0.4, 8.4 Hz, 1H), 7.75 (s, 1H), 7.55 (d, *J* = 1.2 Hz, 1H), 7.33–7.31 (m, 2H), 7.23 (dd, *J* = 2.0, 8.4 Hz, 1H), 7.00 (dd, *J* = 2.4, 9.6 Hz, 1H), 6.94 (dt, *J* = 2.8, 8.4 Hz, 1H), 4.12 (s, 3H), 3.79 (s, 3H), 2.31 (s, 3H), 1.52 (s, 9H); *m/z* (ESI^+^) 550.1 ([M + H]^+^, 100%).

#### *N*-(6-(4-fluoro-2-methylphenyl)-3-(1-methyl-1*H*-indazol-6-yl)imidazo[1,2-*a*]pyridin-8-yl)methanesulfonamide (OXS007564, 6)

To a solution of **22** (120 mg, 0.218 mmol) in 4 M HCl in dioxane (4.0 mL) was added three drops of MeOH and the reaction mixture stirred at room temperature for 3 days. The reaction mixture was then concentrated *in vacuo*, basified with saturated aqueous NaHCO_3_ and extracted with EtOAc. The combined organic layers were concentrated and purified using flash column chromatography (10%–100% EtOAc in hexane), then triturated with Et_2_O and dried to give OXS007564**6** (65 mg, 0.14 mmol, 66%) as an off-white solid. ^1^H NMR (400 MHz, CDCl_3_) *δ* 8.04 (d, *J* = 1.2 Hz, 1H), 8.01 (d, *J* = 1.2 Hz, 1H), 7.88–7.85 (m, 1H), 7.74 (s, 1H), 7.56 (s, 1H), 7.35–7.31 (m, 2H), 7.21 (dd, *J* = 5.6, 8.4 Hz, 1H), 7.02–6.99 (m, 1H), 6.94 (td, *J* = 2.8, 8.4 Hz, 1H), 4.12 (s, 3H), 3.16 (s, 3H), 2.32 (s, 3H); ^13^C NMR (100 MHz, CDCl_3_) *δ* 162.7 (d, *J* = 248 Hz), 140.3, 139.3, 138.6 (d, *J* = 8 Hz)), 133.3 (d, *J* = 3 Hz), 133.1, 132.3, 131.6 (d, *J* = 8 Hz), 128.3, 127.3, 126.6, 126.5, 124.1, 122.4, 120.7, 118.1, 117.5 (d, *J* = 21 Hz), 113.3, 113.2, 113.0, 109.1, 40.2, 35.9, 20.8; *m/z* (ESI^+^) 450.1 ([M + H]^+^, 100%). HRMS (ESI^+^) C_23_H_21_O_2_N_5_FS^+^ ([M + H]^+^) requires 450.1395, found 450.1394.

#### Synthesis of diazirine 29

**Figure:**
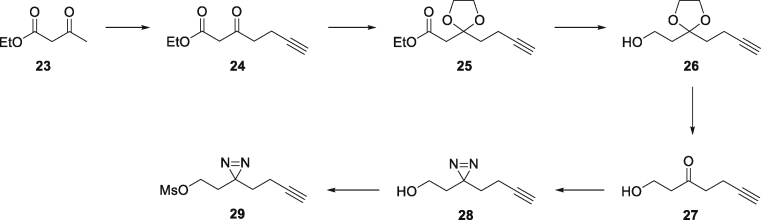

#### Ethyl 3-oxohept-6-ynoate (24)

Lithium diisopropylamide (2 M in THF/heptane/ethylbenzene, 16.9 mL, 33.8 mmol) was added dropwise to a solution of ethyl acetoacetate **23** (1.9 mL, 15 mmol) in THF (15.4 mL) at 0°C under argon. Propargyl bromide (80% w/w, 1.7 mL, 15 mmol) was added dropwise and the reaction was stirred at room temperature for 4 h. The mixture was neutralised with saturated aqueous NH_4_Cl, extracted with EtOAc, dried over anhydrous Na_2_SO_4_ and concentrated *in vacuo*. The crude product was purified using flash column chromatography (5% EtOAc in pentane) to give the product **24** (1.25 g, 7.39 mmol, 48%) as a yellow oil. ^1^H NMR (400 MHz, CDCl_3_) *δ* 4.13 (q, *J* = 7.2 Hz, 2H), 3.41 (s, 2H), 2.77 (t, *J* = 7.1 Hz, 2H), 2.40 (td, *J* = 7.0, 2.7 Hz, 2H), 1.91 (t, *J* = 2.7 Hz, 1H), 1.21 (t, *J* = 7.1 Hz, 3H); ^13^C NMR (100 MHz, CDCl_3_) *δ* 200.6, 166.9, 82.5, 69.0, 61.4, 49.1, 41.5, 14.0, 12.7; *m/z* (ESI^+^) 191.0 ([M + Na]^+^, 100%); HRMS (ESI^+^) C_9_H_13_O_3_^+^ ([M + H]^+^) requires 169.0859, found 169.0861. NMR was in agreement with the reported values (Morcillo et al., 2016).

#### Ethyl 2-(2-(but-3-yn-1-yl)-1,3-dioxolan-2-yl)acetate (25)

BF_3_·OEt_2_ (800 μL, 6.27 mmol) was added dropwise to a solution of **24** (703 mg, 4.18 mmol) and ethylene glycol (900 μL, 16.7 mmol) in CH_2_Cl_2_ (8.4 mL) at 0°C under nitrogen. The reaction was stirred at 0°C for 1 h, before warming to room temperature and stirring overnight. The mixture was cooled to 0°C and water was added dropwise. The crude product was extracted with CH_2_Cl_2_, washed with brine, dried over anhydrous Na_2_SO_4_ and concentrated *in vacuo*. The crude product was purified using flash column chromatography (5% EtOAc in pentane) to give dioxolane **25** (560 mg, 2.63 mmol, 63%) as a yellow oil. ^1^H NMR (400 MHz, CDCl_3_) *δ* 4.15 (q, *J* = 7.1 Hz, 2H), 4.04–3.92 (m, 4H), 2.65 (s, 2H), 2.33–2.26 (m, 2H), 2.15–2.09 (m, 2H), 1.93 (t, *J* = 2.7 Hz, 1H), 1.26 (t, *J* = 7.1 Hz, 3H); ^13^C NMR (100 MHz, CDCl_3_) *δ* 169.3, 108.5, 84.1, 68.2, 65.4, 60.8, 42.8, 36.6, 14.3, 13.0; *m/z* (ESI^+^) 235.0 ([M + Na]^+^, 100%); HRMS (ESI^+^) C_11_H_16_O_4_Na^+^ ([M + Na]^+^) requires 235.0941, found 235.0941. NMR was in agreement with the reported values (Hayakawa et al., 1984).

#### 2-(2-(But-3-yn-1-yl)-1,3-dioxolan-2-yl)ethan-1-ol (26)

A solution of **25** (535 mg, 2.52 mmol) in Et_2_O (12.6 mL) was added dropwise to a solution of LiAlH_4_ (1 M in THF, 2.5 mL, 2.5 mmol) in Et_2_O (15 mL) at 0°C under nitrogen. The reaction was stirred at room temperature for 30 min. The mixture was quenched with a few drops of aqueous 1 M NaOH and water, filtered and concentrated *in vacuo* to give alcohol **26** (314 mg, 1.84 mmol, 73%) as a yellow oil, which was used in the following step without further purification. ^1^H NMR (400 MHz, CDCl_3_) *δ* 4.07–3.93 (m, 4H), 3.76 (q, *J* = 5.7 Hz, 2H), 2.65 (t, *J* = 5.7 Hz, 1H), 2.31–2.23 (m, 2H), 1.96–1.91 (m, 5H); ^13^C NMR (100 MHz, CDCl_3_) *δ* 111.2, 84.1, 68.4, 65.1, 58.9, 38.4, 36.1, 13.3; HRMS (ESI^+^) C_9_H_14_O_3_Na^+^ ([M + Na]^+^) requires 193.0835, found 193.0837. NMR was in agreement with the reported values (Hayakawa et al., 1984).

#### 1-Hydroxyhept-6-yn-3-one (27)

Aqueous HCl (5 M, 7.2 mL, 36 mmol) was added to a solution of **26** (1.44 g, 8.46 mmol) in THF (21.7 mL), and the mixture was stirred at room temperature for 3 h. The mixture was diluted with water, neutralised with aqueous saturated NaHCO_3_ and extracted with EtOAc. The organic layer was dried over anhydrous Na_2_SO_4_ and concentrated *in vacuo* to give deprotected ketone **27** (990 mg, 7.85 mmol, 93%) as a yellow oil. ^1^H NMR (400 MHz, CDCl_3_) *δ* 3.81 (t, *J* = 5.4 Hz, 2H), 2.70–2.56 (m, 4H), 2.41 (td, *J* = 7.2, 2.7 Hz, 2H), 1.89 (t, *J* = 2.7 Hz, 1H); ^13^C NMR (100 MHz, CDCl_3_) *δ* 209.2, 82.9, 69.1, 57.9, 44.7, 41.9, 13.0; HRMS (ESI^+^) C_7_H_10_O_2_Na^+^ ([M + Na]^+^) requires 149.0573, found 149.0573. NMR was in agreement with the reported values (Li et al., 2013).

#### 2-(3-(But-3-yn-1-yl)-3*H*-diazirin-3-yl)ethan-1-ol (28)

Ammonia solution (7 M in MeOH, 3.4 mL, 24 mmol) was added to ketone **27** (200 mg, 1.59 mmol) and stirred for 4.5hat −10°C. A solution of hydroxylamine-*O*-sulfonic acid (233 mg, 2.06 mmol) in dry MeOH (1.2 mL) was then added dropwise at 0°C and stirred for 1 h. The reaction was warmed to room temperature and stirred overnight. Excess ammonia was removed using a stream of nitrogen, and then the mixture was filtered and the solid washed with dry MeOH. Triethylamine (1.0 mL, 12 mmol) was added to the organic phase and cooled to 0°C. Iodine crystals (523 mg, 2.06 mmol) were added portionwise and the mixture was stirred at 0°C for 1 h. The mixture was extracted with Et_2_O, washed with brine and concentrated *in vacuo*. The crude product was purified using flash column chromatography (20% EtOAc in pentane) to give diazirine **28** (35 mg, 0.25 mmol, 16%) as a pale yellow oil. ^1^H NMR (400 MHz, CDCl_3_) *δ* 3.49 (t, *J* = 6.2 Hz, 2H), 2.04 (td, *J* = 7.3, 2.6 Hz, 2H), 2.00 (t, *J* = 2.6 Hz, 1H), 1.74–1.65 (m, 4H); ^13^C NMR (100 MHz, CDCl_3_) *δ* 83.0, 69.4, 57.5, 35.7, 32.8, 26.7, 13.4; *m/z* not found. NMR was in agreement with the reported values (Li et al., 2013).

#### 2-(3-(But-3-yn-1-yl)-3*H*-diazirin-3-yl)ethyl methanesulfonate (29)

Mesyl chloride (23 μL, 0.29 mmol) was added dropwise to a solution of **28** (40 mg, 0.29 mmol) and triethylamine (49 μL, 0.35 mmol) in THF (1.5 mL) at 0 °C under argon, and stirred at room temperature for 2 h. The mixture was diluted with water and extracted with CH_2_Cl_2_. The organic layer was washed with brine, dried with anhydrous Na_2_SO_4_ and concentrated *in vacuo* to give mesyl **29** (53 mg, 0.25 mmol, 85%) as a yellow oil. ^1^H NMR (400 MHz, CDCl_3_) *δ* 4.08 (t, *J* = 6.2Hz, 2H), 3.06 (s, 3H), 2.04 (td, *J* = 7.2, 2.4 Hz, 1H), 2.01 (t, *J* = 2.6 Hz, 1H), 1.90 (t, *J* = 6.2 Hz, 2H), 1.70 (t, *J* = 7.3, 2H); ^13^C NMR (100 MHz, CDCl_3_) *δ* 82.5, 69.7, 64.1, 37.8, 33.0, 32.4, 26.0, 13.5; HRMS (ESI^+^) C_8_H_12_N_2_O_3_SNa^+^ ([M + Na]^+^) requires 239.0461, found 239.0463.

#### Synthesis of probe OXS008450 (4)

**Figure:**
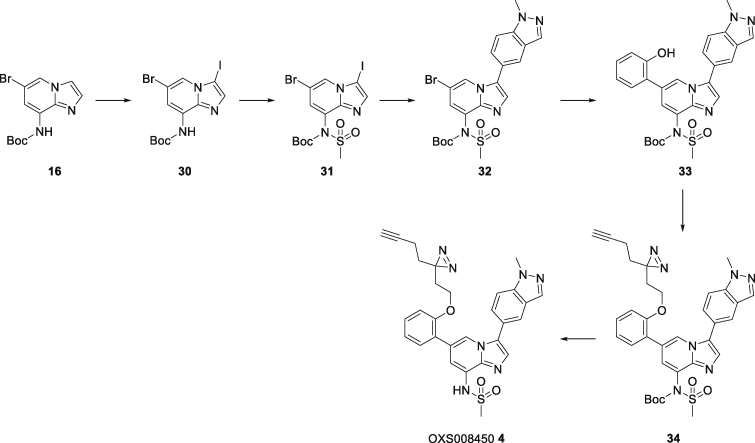

#### *tert*-Butyl (6-bromo-3-iodoimidazo[1,2-*a*]pyridin-8-yl)carbamate (30)

To a stirred solution of **16** (12.7 g, 40.7 mmol) in THF (150 mL) was added *N*-iodosuccinimide (9.15 g, 40.7 mmol), and the mixture was stirred at room temperature for 23 h. The mixture was diluted with water and extracted into CH_2_Cl_2_. The combined extracts were washed with aqueous 1 M Na_2_S_2_O_3_ and brine, dried overMgSO_4_ and concentrated to give a green solid, which was stirred in hexane for 2 h, filtered and dried to give iodide **30** (13.8 g, 31.5 mmol, 78%) as a light green solid. ^1^H NMR (400 MHz, CDCl_3_) *δ* 8.02 (bs, 1H), 7.91 (d, *J* = 1.6 Hz, 1H), 7.83 (bs, 1H), 7.55 (s, 1H), 1.54 (s, 9H); ^13^C NMR (100 MHz, CDCl_3_) *δ* 152.1, 139.8, 138.9, 128.1, 119.4, 112.1, 109.3, 81.8, 62.1, 28.2; *m/z* (ESI^+^) 437.9 ([M + H]^+^, 100%).

#### *tert*-Butyl (6-bromo-3-iodoimidazo[1,2-*a*]pyridin-8-yl)(methylsulfonyl)carbamate (31)

Sodium hydride (60% w/w dispersion in mineral oil, 302 mg, 7.56 mmol) was added portionwise to a solution of iodide **30** (1.10 g, 2.52 mmol) in dry THF (37.6 mL) at 0°C under nitrogen. Mesyl chloride (214 μL, 2.77 mmol) was added dropwise at 0°C. The reaction was stirred at room temperature for 1 h, then diluted with water and the crude product extracted with EtOAc. The organic layer was dried with anhydrous Na_2_SO_4_ and concentrated *in vacuo*. The crude product was purified by trituration with isopropyl alcohol to give mesyl **31** (955 mg, 1.84 mmol, 73%) as a white solid. ^1^H NMR (400 MHz, CDCl_3_) *δ* 8.29 (d, *J* = 1.7 Hz, 1H), 7.66 (s, 1H), 7.46 (d, *J* = 1.7 Hz, 1H), 3.67 (s, 3H), 1.44 (s, 9H); ^13^C NMR (100 MHz, CDCl_3_) *δ* 150.6, 144.2, 141.1, 131.2, 127.2, 125.4, 106.9, 85.9, 62.3, 42.1, 27.9; *m/z* (ESI^+^) 515.5 ([M + H]^+^, 100%); HRMS (ESI^+^) C_13_H_16_^79^BrIN_3_O_4_S^+^ ([M^79^Br + H]^+^) requires 515.9084, found 515.9084.

#### t*ert*-Butyl (6-bromo-3-(1-methyl*-1H-*indazol-5-yl)imidazo[1,2-*a*]pyridin-8-yl)-(methylsulfonyl)carbamate (32)

Pd(dppf)Cl_2_ (7 mg, 0.01 mmol) was added to a stirred solution of **31** (98 mg, 0.19 mmol), (1-methylindazol-5-yl)boronic acid (37 mg, 0.21 mmol) and potassium carbonate (80 mg, 0.58 mmol) in degassed dioxane/water (4:1, 2.0 mL) at room temperature under argon. The resultant mixture was heated to 50°C for 3 h, then cooled to room temperature, diluted with EtOAc, filtered through Celite and concentrated. Purification using flash column chromatography (30%–80% EtOAc in pentane) gave the product **32** (76 mg, 0.15 mmol, 77%) as a yellow solid. ^1^H NMR (400 MHz, CDCl_3_) *δ* 8.36 (d, *J* = 1.7 Hz, 1H), 8.09 (d, *J* = 1.0 Hz, 1H), 7.87 (dd, *J* = 1.6, 0.9 Hz, 1H), 7.65 (s, 1H), 7.58 (dt, *J* = 8.7, 1.0 Hz, 1H), 7.50 (dd, *J* = 8.7, 1.6 Hz, 1H), 7.41 (d, *J* = 1.7 Hz, 1H), 4.15 (s, 3H), 3.75 (s, 3H), 1.50 (s, 9H); ^13^C NMR (100 MHz, CDCl_3_) *δ* 150.8, 142.1, 139.8, 133.4, 133.2, 130.5, 127.5, 126.9, 125.7, 124.6, 124.4, 121.7, 120.4, 110.4, 106.1, 85.7, 42.2, 35.9, 28.0; HRMS (ESI^+^) C_21_H_23_^79^BrN_5_O_4_S^+^ ([M^79^Br + H]^+^) requires 520.0649, found 520.0646.

#### *tert*-Butyl (6-(2-hydroxyphenyl)-3-(1-methyl-1H-indazol-5-yl)imidazo[1,2-*a*]-pyridin-8-yl)(methylsulfonyl)carbamate (33)

Pd(dppf)Cl_2_ (34 mg, 0.046 mmol) was added to a stirred solution of **32** (398 mg, 0.764 mmol), 2-hydroxyphenylboronic acid (105 mg, 0.764 mmol) and potassium phosphate tribasic (568 mg, 2.68 mmol) in degassed DME/water (2.3:1, 5 mL) at room temperature under argon. The resultant mixture was heated to 60°C for 1 h, then cooled to room temperature, diluted with EtOAc, filtered through Celite and concentrated. Purification using flash column chromatography (80% EtOAc in pentane) gave the product **33** (389 mg, 0.729 mmol, 95%) as an orange solid. ^1^H NMR (400 MHz, CDCl_3_) *δ* 8.65 (d, *J* = 1.5 Hz, 1H), 7.92 (d, *J* = 0.6 Hz, 1H), 7.84 (dd, *J* = 1.2, 0.3 Hz, 1H), 7.64 (s, 1H), 7.60 (d, *J* = 1.4 Hz, 1H), 7.52–7.49 (m, 2H), 7.33 (dd, *J* = 7.6, 1.7 Hz, 1H), 7.20 (ddd, *J* = 8.0, 7.4, 1.7 Hz, 1H), 6.99–6.90 (m, 2H), 4.08 (s, 3H), 3.71 (s, 3H), 1.49 (s, 9H); ^13^C NMR (100 MHz, CDCl_3_) *δ* 153.8, 151.1, 142.5, 139.7, 133.2, 132.5, 130.5, 130.1, 129.8, 127.4, 127.4, 124.6, 124.5, 124.2, 123.4, 123.2, 121.4, 121.1, 121.0, 116.8, 110.2, 85.7, 42.4, 35.8, 28.0; *m/z* (ESI^+^) 534.2 ([M + H]^+^, 100%); HRMS (ESI^+^) C_27_H_28_N_5_O_5_S^+^ ([M + H]^+^) requires 534.1806, found 534.1799.

#### *tert*-Butyl (3-(1-methyl-1*H*-indazol-5-yl)-6-(2-(2-(3-(prop-2-yn-1-yl)-3*H*-diazirin-3-yl)ethoxy)phenyl)imidazo[1,2-*a*]pyridin-8-yl)(methylsulfonyl)carbamate (34)

A solution of mesyl **29** (24 mg, 0.11 mmol) in dry DMF (0.5 mL) was added dropwise to a solution of phenol **33** (50 mg, 0.094 mmol) and potassium carbonate (16 mg, 0.11 mmol) in dry DMF (1.0 mL) at 0°C under nitrogen, and the mixture was stirred at 60°C for 30 h. The mixture was diluted with water, extracted with EtOAc, dried over anhydrous Na_2_SO_4_ and concentrated *in vacuo* to give the product **34** (36 mg, 0.056 mmol, 60%) as a white solid, which was used in the following step without further purification. ^1^H NMR (400 MHz, CDCl_3_) *δ* 8.47 (d, *J* = 1.5 Hz, 1H), 8.06 (s, 1H), 7.92 (s, 1H), 7.66 (s, 1H), 7.63 (d, *J* = 1.5 Hz, 1H), 7.61–7.50 (m, 2H), 7.36–7.29 (m, 2H), 7.02 (td, *J* = 7.5, 0.7 Hz, 1H), 6.90 (dd, *J* = 8.4, 0.7 Hz, 1H), 4.14 (s, 3H), 3.78 (s, 3H), 3.74 (t, *J* = 6.3 Hz, 1H), 1.87–1.75 (m, 5H), 1.52 (s, 9H), 1.46 (t, *J* = 7.4 Hz, 1H); ^13^C NMR (100 MHz, CDCl_3_) *δ* 155.5, 151.1, 142.4, 139.6, 133.2, 132.6, 130.7, 130.4, 129.7, 127.22, 127.16, 125.8, 124.5, 124.2, 123.47, 123.45, 121.44, 121.36, 121.3, 112.2, 110.0, 85.1, 82.7, 69.0, 62.9, 42.1, 35.8, 32.4, 32.3, 27.9, 13.0; HRMS (ESI^+^) C_34_H_36_N_7_O_5_S^+^ ([M + H]^+^) requires 654.2493, found 654.2481.

#### *N*-(3-(1-methyl-1*H*-indazol-5-yl)-6-(2-(2-(3-(prop-2-yn-1-yl)-3*H*-diazirin-3-yl)-ethoxy)phenyl)imidazo[1,2-*a*]pyridin-8-yl)methanesulfonamide (OXS008450, 4)

A solution of Boc-protected **34** (32 mg, 0.048 mmol) and trifluoroacetic acid (185 μL, 2.42 mmol) in CH_2_Cl_2_ (1.0 mL) was stirred at room temperature under nitrogen for 3 h. The reaction mixture was neutralised with saturated NaHCO_3_, extracted with three times with CH_2_Cl_2_ and washed with saturated aqueous NH_4_Cl. The organic layer was dried with anhydrous Na_2_SO_4_, filtered and concentrated *in vacuo* to obtain the deprotected product OXS008450**4** (24 mg, 0.044 mmol, 91%) as a beige solid. ^1^H NMR (400 MHz, CDCl_3_) *δ* 8.26 (d, *J* = 1.0 Hz, 1H), 8.06 (s, 1H), 7.93 (s, 1H), 7.67 (s, 1H), 7.64 (d, *J* = 1.0 Hz, 1H), 7.61–7.51 (m, 2H), 7.38–7.29 (m, 2H), 7.03 (td, *J* = 7.5, 0.6 Hz, 1H), 6.93 (dd, *J* = 8.2, 0.7 Hz, 1H), 4.14 (s, 3H), 3.83 (t, *J* = 6.2 Hz, 2H), 3.17 (s, 3H), 1.82–1.70 (m, 5H), 1.45 (t, *J* = 7.5 Hz, 2H); ^13^C NMR (126 MHz, CDCl_3_) *δ* 155.7, 139.7, 139.3, 133.3, 131.6, 131.0, 129.8, 128.2, 127.2, 126.5, 125.9, 124.7, 124.6, 121.5, 121.4, 121.2, 118.9, 114.5, 112.2, 110.1, 82.5, 69.1, 63.1, 40.1, 35.9, 32.8, 32.5, 26.6, 13.1; *m/z* (ESI^+^) 554.2 ([M + H]^+^, 100%); HRMS (ESI^+^) C_29_H_28_N_7_O_3_S^+^ ([M + H]^+^) requires 554.1969, found 554.1969.

#### Synthesis of OXS008255 (5)

**Figure:**
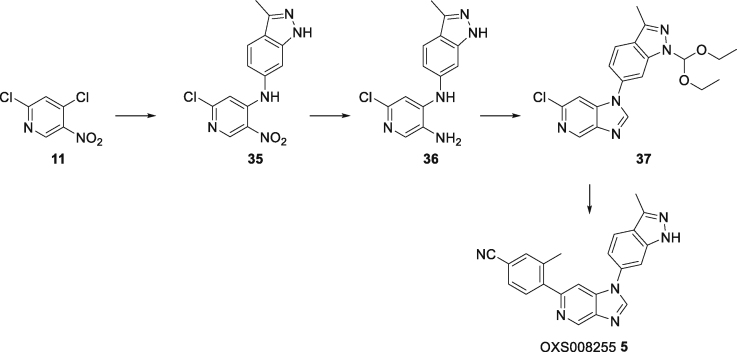

#### *N*-(2-chloro-5-nitropyridin-4-yl)-3-methyl-1*H*-indazol-6-amine (35)

2,4-Dichloro-5-nitro-pyridine **11** (500 mg, 2.59 mmol) and 3-methyl-1*H*-indazol-6-amine (381 mg, 2.59 mmol) were dissolved in MeCN (10 mL), triethylamine (722 μL, 5.18 mmol) was added and the mixture stirred at room temperature for 3 days. The mixture was concentrated *in vacuo*, water was added and the resulting precipitate filtered off, and washed with water and Et_2_O to give the product **35** (918 mg, 3.02 mmol, quant.) as an orange solid. ^1^H NMR (400 MHz, DMSO-*d*_6_) *δ* 12.74 (bs, 1H), 10.02 (s, 1H), 8.97 (s, 1H), 7.81 (d, *J* = 8.8 Hz, 1H), 7.46 (s, 1H), 7.06 (dd, *J* = 8.4, 1.6 Hz, 1H), 6.78 (s, 1H), 2.51 (s, 3H); ^13^C NMR (100 MHz, DMSO-*d*_6_) *δ* 155.0, 149.3, 149.2, 141.9, 141.7, 135.4, 130.8, 121.8, 121.6, 118.5, 108.9, 107.6, 12.1; *m/z* (ESI^+^) 304.0 ([M + H]^+^, 100%).

#### 6-Chloro-*N*^4^-(3-methyl-1*H*-indazol-6-yl)pyridine-3,4-diamine (36)

Nitro compound **35** (303 mg, 2.47 mmol) in IMS (40 mL) was placed in an autoclave. Pd/C (10%, 131 mg, 0.123 mmol) was added and the reaction was heated to 25°Cat 10 bar H_2_ for 3.5 h. The mixture was filtered through Celite and concentrated *in vacuo*. The residue was purified using flash column chromatography (0%–100% EtOAc in hexane) to give amine **36** (450 mg, 1.64 mmol, 66%) as an orange solid. ^1^H NMR (400 MHz, DMSO-*d*_6_) *δ* 12.35 (s, 1H), 7.82 (s, 1H), 7.50 (dd, *J* = 8.4 Hz, 1H), 7.65 (s, 1H), 7.14 (d, *J* = 1.6 Hz, 1H), 6.94–6.91 (m, 1H), 6.83 (s, 1H), 5.03 (s, 2H), 2.45 (s, 3H); ^13^C NMR (100 MHz, DMSO-*d*_6_) *δ* 142.2, 141.6, 140.6, 139.5, 139.1, 135.0, 133.5, 121.3, 119.2, 115.5, 107.1, 100.2, 12.1; *m/z* (ESI^+^) 274.1 ([M + H]^+^, 100%).

#### 6-Chloro-1-(1-(diethoxymethyl)-3-methyl-1*H*-indazol-6-yl)-1*H*-imidazo[4,5-*c*]pyridine (37)

Compound **36** (450 mg, 1.64 mmol) was dissolved in diethoxymethoxyethane (4.87 g, 32.9 mol), formic acid (62 μL, 1.6 mmol) was added and the mixture heated to 100°C overnight. The mixture was cooled and concentrated *in vacuo*. Saturated aqueous NaHCO_3_ was added, and the resulting precipitate was filtered off, and washed with water and hexane to give the cyclised product **37** (420 mg, 1.09 mmol, 66%) as a light brown solid.^1^H NMR (400 MHz, DMSO-*d*_6_) *δ* 8.92 (d, *J* = 0.8 Hz, 1H), 8.83 (s, 1H), 8.00 (d, *J* = 8.4 Hz, 1H), 7.97 (d, *J* = 1.2 Hz, 1H), 7.61 (d, *J* = 0.8 Hz, 1H), 7.50 (dd, *J* = 2.0, 8.4 Hz, 1H), 6.52 (s, 1H), 3.75‒3.68 (m, 2H), 3.60‒3.49 (m, 2H), 2.57 (s, 3H), 1.18‒1.14 (m, 6H); ^13^C NMR (100 MHz, DMSO-*d*_6_) *δ* 147.2, 143.8, 142.8, 142.3, 141.2, 141.0, 139.1, 133.8, 124.2, 122.9, 117.8, 107.0, 106.3, 105.9, 62.4, 15.2, 12.0; *m/z* (ESI^+^) 284.1 ([M + H]^+^, 100%).

#### 3-Methyl-4-[1-(3-methyl-1*H*-indazol-6-yl)imidazo[4,5-*c*]pyridin-6-yl]benzonitrile (OXS008255, 5)

To a solution of **37** (75 mg, 0.19 mmol) in degassed 1,4-dioxane/water (4:1, 1.0 mL) were added potassium phosphate tribasic (121 mg, 0.570 mmol), (4-cyano-2-methylphenyl)boronic acid (37 mg, 0.23 mmol) and Pd(dppf)Cl_2_ (15 mg, 0.020 mmol). The mixture was heated at 100°C overnight, then diluted with EtOAc, filtered through Celite and concentrated. To a solution of the resulting material (30 mg) in ethanol (1.2 mL) was added aqueous HCl (2 M, 110 μL, 0.21 mmol) at 0°C. After stirring for 30minat room temperature, the mixture was basified with aqueous 2 M NaOH. After a further 30 min, water was added and the mixture was extracted with EtOAc. The organic layer was dried over anhydrous Na_2_SO_4_, evaporated and purified using flash column chromatography (EtOAc) to give OXS008255**5** (5 mg, 0.01 mmol, 7%). ^1^H NMR (500 MHz, CDCl_3_) *δ* 9.32 (d, *J* = 1.1 Hz, 1H), 8.33 (s, 1H), 7.93 (d, *J* = 8.4 Hz, 1H), 7.64–7.57 (m, 3H), 7.57–7.50 (m, 2H), 7.32 (dd, *J* = 8.4, 1.8 Hz, 1H), 2.69 (s, 3H), 2.43 (s, 3H); ^13^C NMR (126 MHz, CDCl_3_) *δ* 152.2, 145.3, 144.5, 144.1, 143.1, 141.1, 140.3, 139.3, 137.8, 134.3, 133.8, 130.7, 129.6, 122.9, 122.6, 118.9, 116.8, 111.8, 106.0, 105.3, 20.4, 12.0; *m/z* (ESI^+^) 365.1 ([M + H]^+^, 100%); HRMS (ESI^+^) C_22_H_17_N_6_^+^ ([M + H]^+^) requires 365.1509; found 365.1512.

### Quantification and statistical analysis

#### Gene expression analysis

Following sequencing, QC analysis was conducted using the fastQC package (http://www.bioinformatics.babraham.ac.uk/projects/fastqc). Reads were mapped to the human genome assembly hg19 using STAR (Dobin and Gingeras, 2015). The featureCounts function from the Subread package was used to quantify gene expression levels using standard parameters (Liao et al., 2019). This was used to identify differential gene expression globally, using the DESeq2 package (Love et al., 2014).

#### Proteomics database search and data analysis

Processing of LC-MS/MS data was performed in MaxQuant version 1.6.6.0 (Cox and Mann, 2008) using the built-in Andromeda search engine. Peptides were identified from the MS/MS spectra searched against the human reference proteome (Uniprot, Taxon ID: 9606, accessed 4th September 2019). Cysteine carbamidomethylation was set as a fixed modification, and methionine oxidation and N-terminal acetylation were set as variable modifications. ‘Trypsin/P’ was chosen as digestion mode enzyme. Minimum peptide length was set to 7 residues and maximum 2 missed cleavages were allowed. ‘Unique and razor peptides’ were chosen for protein quantification. Quantification parameters were set to ‘standard’ and ‘LFQ’. Other parameters were used as pre-set in the software.

Data analysis was performed using Perseus (version 1.6.6.0) (Tyanova et al., 2016). MaxQuant proteinGroups.txt output files were filtered against contaminants and reverse dataset. Base 2 logarithm was applied to all measurements and the median values within each sample were subtracted to normalise for sample variation associated with overall protein abundance. The replicates for each condition were grouped and the proteins with at least two valid values within a group were kept. A student’s t-test (FDR = 0.05; S0 = 0.1) was performed between the active probe sample and each DMSO control, and between active probe sample and probe/parent competition samples. For mathematical reasons (Giai Gianetto et al., 2016), S_0_ was kept low. The results were plotted using GraphPad Prism.

#### Statistical analysis

Details of tests used, n values and p values are provided in figure legends or STAR Methods.

## Data Availability

• Processed proteomics data are available in Tables S3 and S4–S7. The raw mass spectrometry proteomics files and database search results have been deposited at the ProteomeXchange Consortium (http://proteomecentral.proteomexchange.org) via the PRIDE partner repository (Perez-Riverol et al., 2019) with dataset identifier PXD0022038 and are publicly available as of the date of publication. Sequencing data that support the findings of this study have been deposited in the Gene Expression Omnibus (GEO) with the accession code GSE178787. https://www.ncbi.nlm.nih.gov/geo/query/acc.cgi?acc=GSE178787 and are publicly available as of the date of publication. This paper also analyzes existing, publicly available data. These accession numbers for the datasets are listed in the key resources table. Original western blot image is available in Figure S5. Microscopy and Flow Cytometry data reported in this paper will be shared by the lead contact upon request. • This paper does not report original code. • Any additional information required to reanalyze the data reported in this paper is available from the lead contact upon request.

## Supplemental information

Document S1. Figures S1–S6 and Tables S1, S2, and S4–S7

Table S3. Significantly enriched proteins by probe 4 compared to DMSO, full dataset in Table S8, related to Figure 6

Table S8. Full dataset of significantly enriched proteins, related to Figure 6
