# Supplementary material for: A tubulin binding molecule drives differentiation of acute myeloid leukemia cells
Source: iScience. 2022 Jul 19;25(8):104787. doi: 10.1016/j.isci.2022.104787 (PMC9385704; doi:10.1016/j.isci.2022.104787)
Supplement: Document S1. Figures S1–S6 and Tables S1, S2, and S4–S7 [file mmc1.pdf]

iScience, Volume *25*

Supplemental information

A tubulin binding molecule drives differentiation of acute myeloid leukemia cells

Thomas R. Jackson, Aini Vuorinen, Laia Josa-Culleré, Katrina S. Madden, Daniel Conole, Thomas J. Cogswell, Isabel V.L. Wilkinson, Laura M. Kettyle, Douzi Zhang, Alison O'Mahony, Deanne Gracias, Lorna McCall, Robert Westwood, Georg C. Terstappen, Stephen G. Davies, Edward W. Tate, Graham M. Wynne, Paresh Vyas, Angela J. Russell, and Thomas A. Milne

Figure S1. High-Throughput screening in multiple AML cell lines, related to Figure 1. (A) Upon treatment with positive control, PMA, KG1 cells differentiate and up-regulate sell surface marker CD11b as detected by flow cytometry (left). Scatter plot distribution showing the results of HL-60 high-throughput screening of 1000 compound library (right). (B) Upon treatment with positive control, GS87 (30μM), OCI-AML3 cells differentiate and up-regulate sell surface marker CD11b as detected by flow cytometry (left). Scatter plot distribution showing the results of HL-60 high-throughput screening of 1000 compound library (right). (C) Upon treatment with positive control, TCP, THP-1 cells differentiate and up-regulate sell surface marker CD11b as detected by flow cytometry (left). Scatter plot distribution showing the results of HL-60 high-throughput screening of 1000 compound library (right).

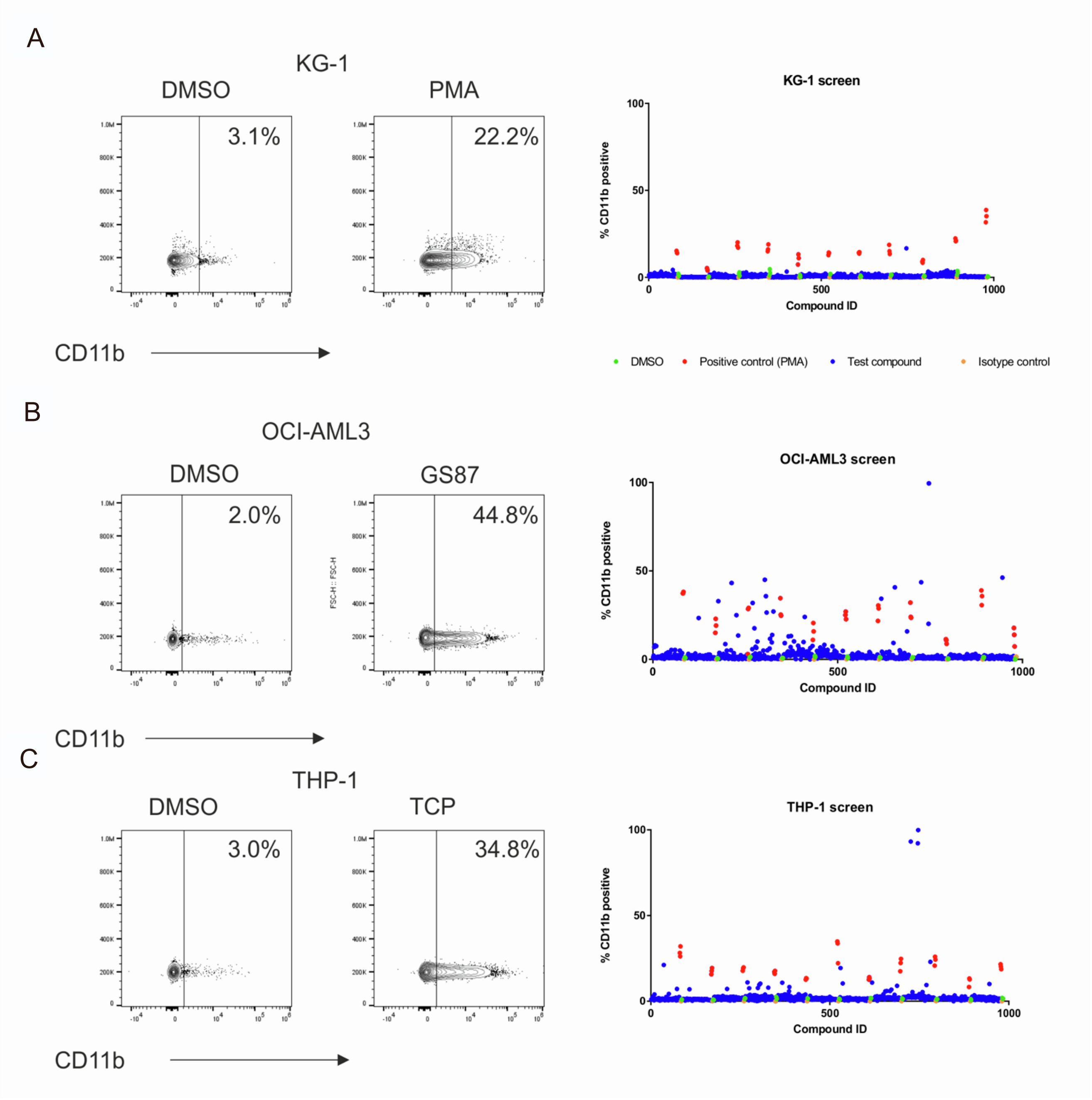

Figure S2. Macrophage-like morphology observed in AML cell lines treated with OXS000275, related to Figure 1. Cytospin preparations of OXS000275-treated cells lines stained with Wright-Giemsa showed signs of myeloid maturation.

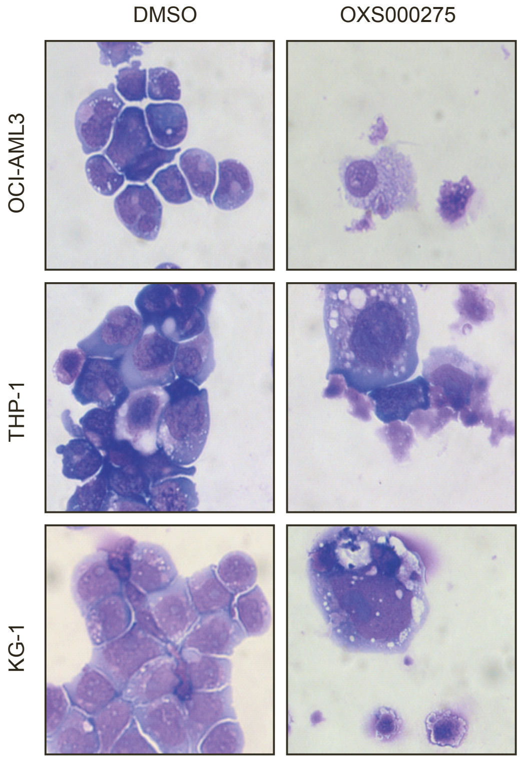

Figure S3. OXS007464 and OXS007417 treatment leads to an increase in absolute number of CD11b positive cells, related to Figure 3. Cells were treated with 10mM of OXS007464 or OXS007417 for 24 hours. Absolute number of CD11b cells were determined by flow cytometry. Error bars = SEM, * $p < 0.05$.

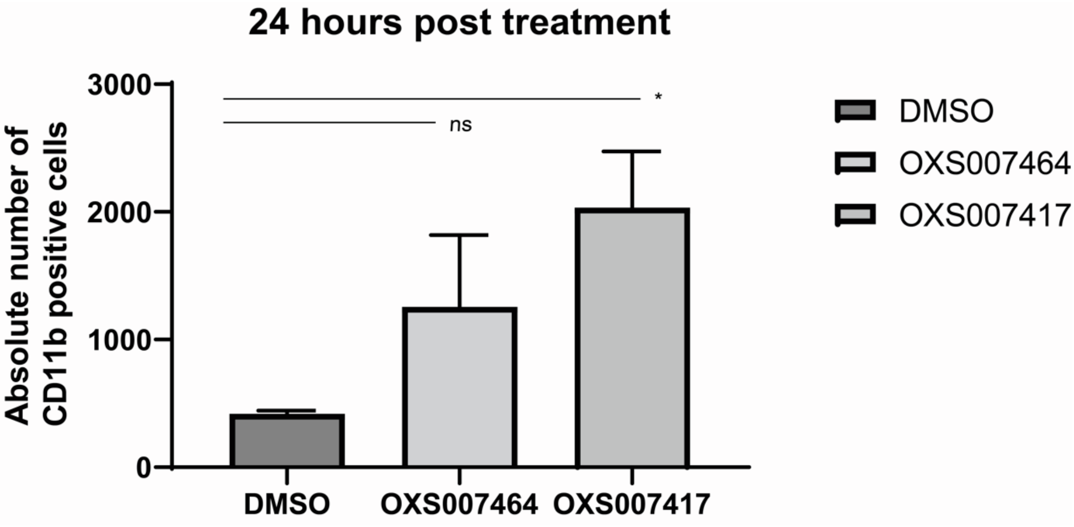

Figure S4. Normalized body weight or Subcutaneous Xenograft Model mice over time with indicated treatments, related to Figure 4. Error bars represent SD.

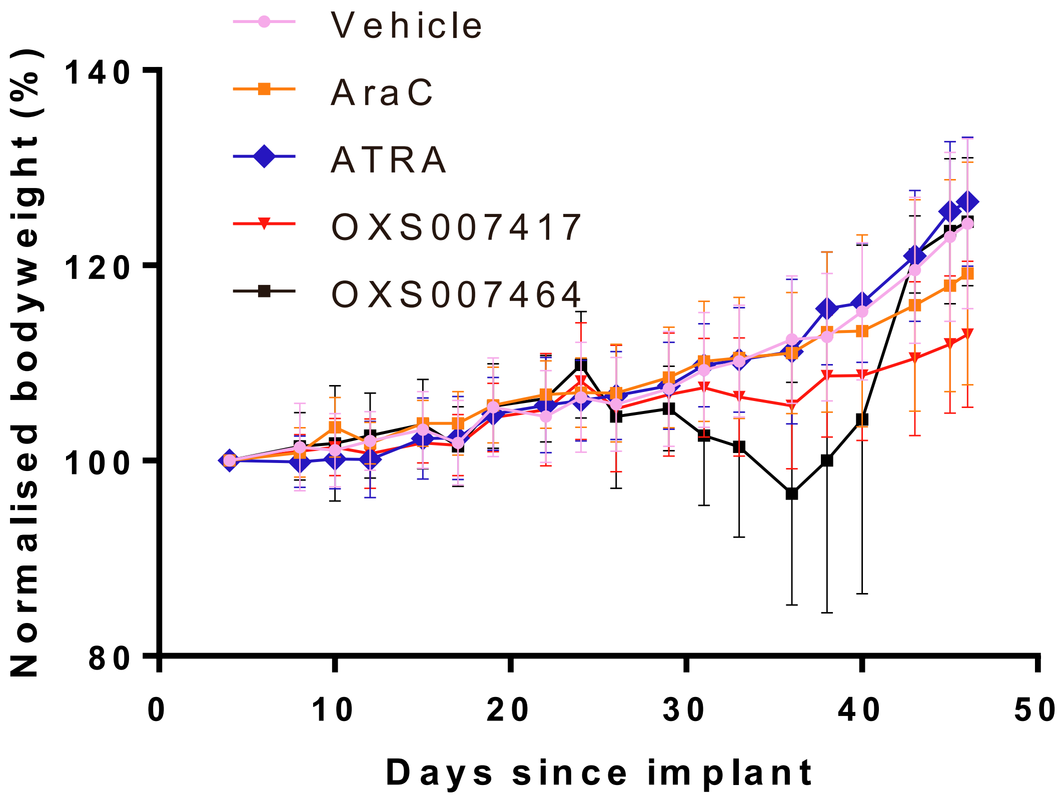

Figure S5. Control gels and data for Figure 6, related to Figure 6.

(A) In-gel fluorescence showing non-irradiated control and competition of OXS007464 **3** and OXS007564 **6** with probe **4.** (B) Structure of OXS007564 **6**. EC50 > 10 µM. (C) Structure of AzRB capture reagent. (PMID: 25807930). (D) Volcano plot showing significantly enriched proteins in the pull-down experiment by probe **4** compared to DMSO vehicle. (E) Uncropped western blot from Figure 6I.

A

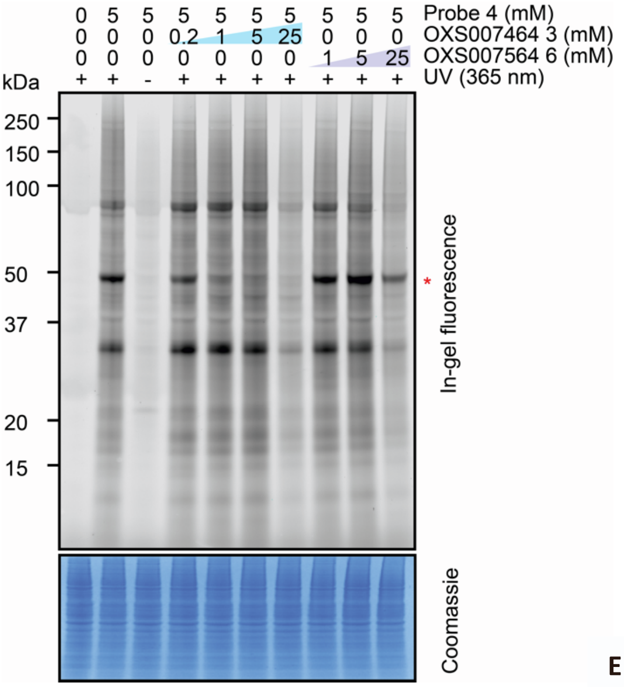

D

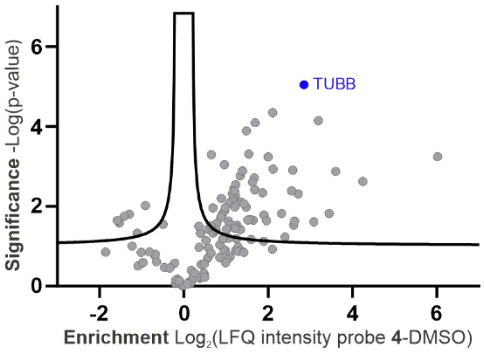

E

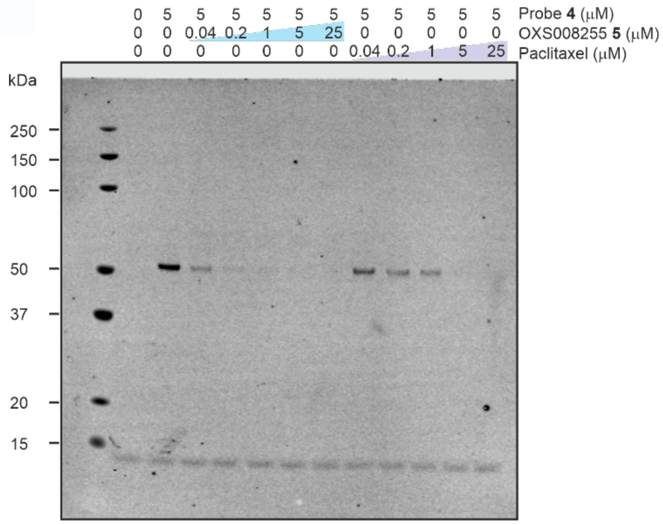

B

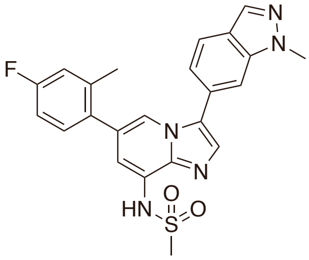

OXS007564 **3**
EC$_{50}$ >10 µM

C

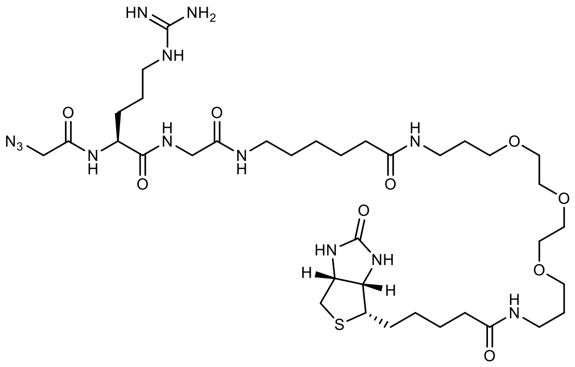

Figure S6. **Tubulin disrupters with diverse structures cause upregulation of CD11b, related to Figure 8. (A)** Treatment of HL-60 cells with Nocodazole lead to an upregulation of CD11b in a dose dependent manner when measured by flow cytometry. **(B)** Dose response curve of CD11b upregulation and cell viability in response to Nocodazole as determined by flow cytometry**.** Two technical replicates were performed per experiment with n=2 biological replicates. One representative example curve is shown. **(C)** Treatment of HL-60 cells with ABT-751 lead to an upregulation of CD11b in a dose dependent manner when measured by flow cytometry. **(D)** Dose response curve of CD11b upregulation and cell viability in response to ABT-751 as determined by flow cytometry**.** Two technical replicates were performed per experiment with n=2 biological replicates. One representative example curve is shown. **(E)** Treatment of HL-60 cells with CYT997 lead to an upregulation of CD11b in a dose dependent manner when measured by flow cytometry. **(F)** Dose response curve of CD11b upregulation and cell viability in response to CYT997 as determined by flow cytometry**.** Two technical replicates were performed per experiment with n=2 biological replicates. One representative example curve is shown.

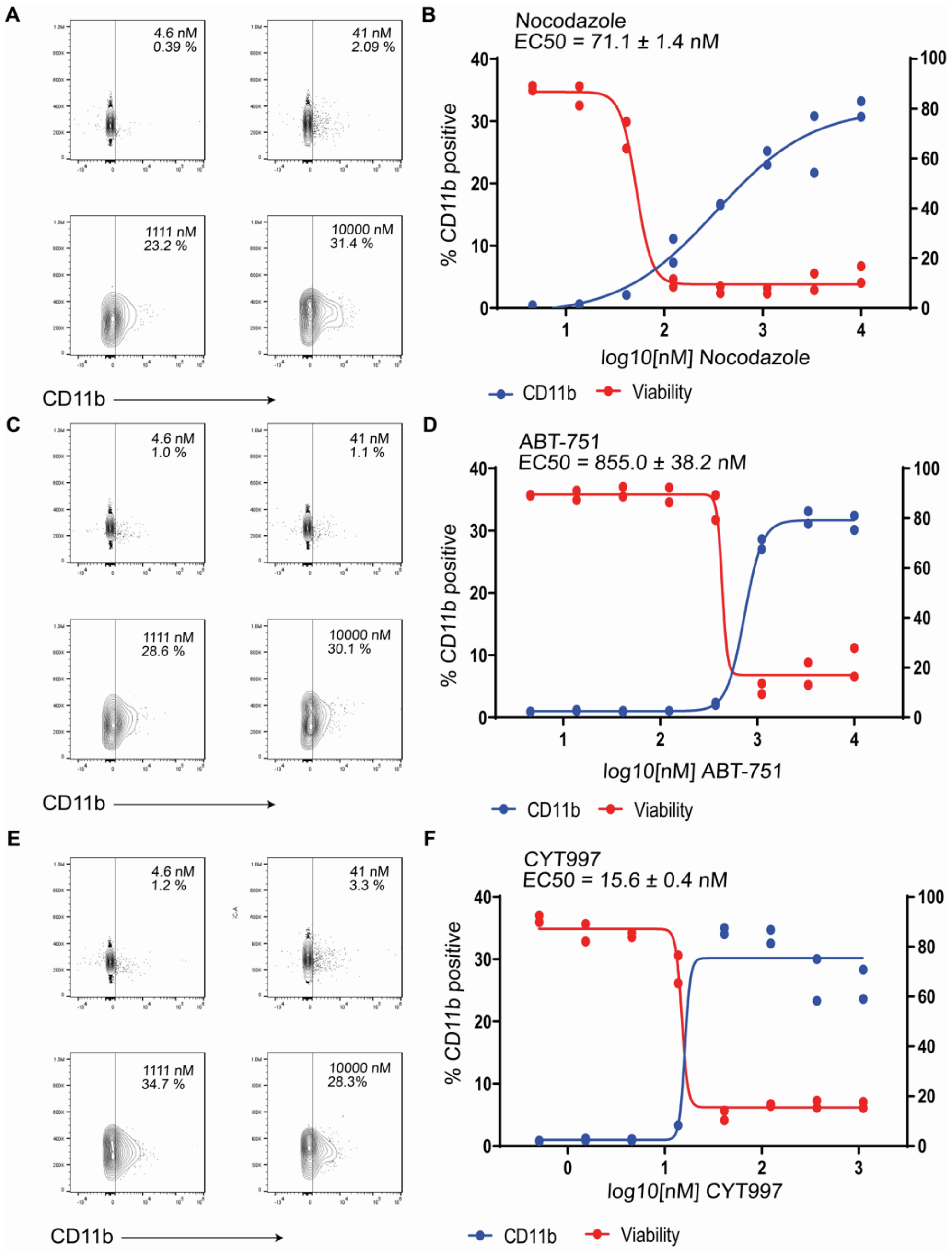

Table S1. Compound screening data. Related to Figure 1, S1 and STAR Methods.

Category	Parameter	Description
Assay	Type of assay	*In vitro*, cell-based, antibody detection
	Target	CD11b
	Primary measurement	Detection of CD11b expression with flow cytometry
	Key reagents	Anti-human CD11b/Mac-1 (555388, BDBioscience), DAPI (D9542, Sigma-Aldrich),
	Assay protocol	Cells were pelleted by centrifugation at 1000 rpm and suspended in 40 mL of blocking buffer (10% FBS in IMDM, no phenol red), then 10 mL of anti-human CD11b/Mac-1 solution (25% in blocking buffer) was added. Cells were stored in ice for 20 min. The cell suspension was centrifuged, washed three times with staining buffer (1% FBS in IMDM, no phenol red), and resuspended in 200 mL of staining buffer with 1 mg/mL DAPI. Flow cytometry was performed on an Attune NxT flow cytometer (Thermo Fisher Scientific UK) with previous compensation.
	Additional comments	4 cell lines: HL-60, OCI-AML3, THP-1, KG-1, purchased from ATCC or form DSMZ
Library	Library size	1000 compounds
	Library composition	Small drug-like molecules, selected from a bigger library of 6991 molecules based on structural diversity. These were selected with the “Select Diverse Set” function of Datawarrior; those 96-well plates having the highest number of compounds with high Diversity Selection Rank scores were selected for testing.
	Source	BioFocus‘SoftFocus’ collection
	Additional comments	
Screen	Format	96-well plates
	Concentration(s) tested	10 μM, 0.1% DMSO
	Plate controls	HL-60 and KG-1: 0.1% DMSO (negative control); 10 nM PMA (positive control); 10 nM PMA stained with IgG isotype instead of CD11b<br>OCI-AML3: : 0.1% DMSO (negative control); 30 μM GS87 (positive control); 30 μM GS87 stained with IgG isotype instead of CD11b<br>THP-1: 0.1% DMSO (negative control); 10 μM tranylcypromine (positive control); 10 μM tranylcypromine stained with IgG isotype instead of CD11b
	Reagent/ compound dispensing system	Manual pipetting
	Detection instrument and software	Attune NxT flow cytometer (Thermo Fisher Scientific UK)
	Assay validation/QC	Before the HTS, a smaller pilot screen was performed to confirm correlation between CD11b expression and cell differentiation (assessed by morphology and proliferation).

Z’ factor and signal to background (S/B) were analysed across all tested plates:

	Z’ factor	S/B
HL-60	0.90 ±0.12	120 ±80
OCI-AML3	0.55 ±0.22	24 ±5
THP-1	0.64 ±0.27	15 ±7
KG-1	0.79 ±0.16	11 ±4

Each individual plate was only analysed if negative control was <2% CD11b and positive control was >60% (HL-60). >10% (THP-1, KG-1), or >20% (OCI-AML3)

	Correction factors	Gating of the CD11b negative population was performed on the negative and non-stained control wells, and the same gating was maintained for all test compounds.
	Normalization	NA
	Additional comments	
Post-HTS analysis	Hit criteria	>10% CD11b
	Hit rate	44 hits
	Additional assay(s)	Isotype control to rule out fluorescent compounds and/or non-specific antibody binding. Cell morphology, cell proliferation (cell number and viability with acridin orange and PI), retesting the resynthesised compounds in dose-response in the CD11b assay
	Confirmation of hit purity and structure	Compounds were resynthesized and fully analyzed to enable unambiguous identification
	Additional comments	

Table S2. Summary of EnrichR analysis of RNA-seq signatures for a range in chemically distinct original hits. Related to Figure 2.

Compound	FACS %CD11b	log2foldchange CD11b	Total up	Total down	Macrophage No. up	Macrophage % of up	Fisher exact p-value	Neutrophil No. up	Neutrophil % of up	Fisher exact p-value
OXS000651	15	1.5	1241	973	294	23.69057	1.92E-42	299	24.09347	1.01E-44
OXS006988	16	1.8	2704	2382	301	11.13166	1.02E-08	282	10.42899	0.001
OXS006996	42	2.8	2544	2312	420	16.50943	3.45E-22	517	20.32233	3.91E-54
OXS003976	72.5	3.4	4183	3717	1429	34.16208	NA	724	17.30815	2.7E-49
OXS006976	20	2.3	2827	2434	343	12.133	0.005964	333	11.77927	0.033432
OXS004030	58	4.4	3384	3144	700	20.68558	7.8E-251	504	14.89362	3.12E-15
OXS000493	25	2	2869	2714	260	9.062391	0.86	248	8.644127	0.8
OXS000366	20	1.3	1395	1380	161	11.54122	0.243117	150	10.75269	2E-12
PMA	80	5.4	4255	3738	748	17.57932	2.64E-54	504	11.84489	0.004274
ATRA	24	3.4	3133	2753	808	25.78998	9.9E-156	571	18.22534	4.2E-44
OXS006974 (inactive control)		0.355725	45	0	3	6.666667	0.624736	0	0	NA

Table S4. Significantly enriched proteins by probe **4** compared to competition with OXS008255 **5** 1 µM, full dataset in Table S8. Related to Figure 6.

Gene names	Protein names	Majority protein IDs	Peptides	Razor + unique peptides	Unique peptides	Sequence coverage [%]	Unique + razor sequence coverage [%]	Unique sequence coverage [%]	Mol. weight [kDa]	Score	Intensity	MS/MS count	Normalised Log_2(LFQ intensity)					
													OXS008450			OXS008450/OXS008255 1 µM		
													1	2	3	1	2	3
TUBB; TUBB2B; TUBB2A	Tubulin beta chain; Tubulin beta-2B chain; Tubulin beta-2A chain	Q5JP53; P07437; Q5ST81; Q9BVA1; Q13885	24	24	4	59.2	59.2	10.6	47.77	323	1.7E+11	833	5.7159	5.6001	5.8671	4.8092	4.6922	4.2793

Table S5. Significantly enriched proteins by probe **4** compared to competition with OXS008255 **5** 5 µM, full dataset in Table S8. Related to Figure 6.

Gene names	Protein names	Majority protein IDs	Peptides	Razor + unique peptides	Unique peptides	Sequence coverage [%]	Unique + razor sequence coverage [%]	Unique sequence coverage [%]	Mol. weight [kDa]	Score	Intensity	MS/MS count	Normalised Log_2(LFQ intensity)					
													OXS008450			OXS008450/OXS008255 5 µM		
													1	2	3	1	2	3
TUBB; TUBB2B; TUBB2A	Tubulin beta chain; Tubulin beta-2B chain; Tubulin beta-2A chain	Q5JP53; P07437; Q5ST81; Q9BVA1; Q13885	24	24	4	59.2	59.2	10.6	47.77	323	1.7E+11	833	5.7159	5.6001	5.8671	4.6937	4.7750	4.7751
YWHAG	14-3-3 protein gamma; 14-3-3 protein gamma, N-terminally processed	P61981	10	6	6	39.7	25.9	25.9	28.3	60.5	2.1E+09	52	NaN	-0.6701	-0.2213	-1.6246	-1.6044	-1.1383

Table S6. Significantly enriched proteins by probe **4** compared to competition with OXS008255 **5** 25 µM, full dataset in Table S8. Related to Figure 6.

Gene names	Protein names	Majority protein IDs	Peptides	Razor + unique peptides	Unique peptides	Sequence coverage [%]	Unique + razor sequence coverage [%]	Unique sequence coverage [%]	Mol. weight [kDa]	Score	Intensity	MS/MS count	Normalised Log_2(LFQ intensity)					
													OXS008450			OXS008450/OXS008255 25 µM		
													1	2	3	1	2	3
TUBB; TUBB2B; TUBB2A	Tubulin beta chain; Tubulin beta-2B chain; Tubulin beta-2A chain	Q5JP53; P07437; Q5ST81; Q9BVA1; Q13885	24	24	4	59.2	59.2	10.6	47.77	323	1.7E+11	833	5.7159	5.6001	5.8671	4.2771	5.0476	4.3656
DCTPP1	dCTP pyrophosphatase 1	Q9H773	5	5	5	39.4	39.4	39.4	18.68	78.8	5.2E+09	61	1.0178	0.7472	0.7494	-0.4114	-0.6164	-0.4911
TRAM1	Translocating chain-associated membrane protein 1	G3XAN4; Q15629	8	8	8	24.7	24.7	24.7	33.44	89.6	2.3E+09	50	-0.1936	-0.1782	-0.1459	NaN	-1.0706	-1.4249
SRPRB	Signal recognition particle receptor subunit beta	Q9Y5M8	7	7	7	31.4	31.4	31.4	29.7	45.9	5E+09	58	0.3632	0.2234	0.2824	-0.5229	-0.6527	-0.7863
FKBP1A	Peptidyl-prolyl cis-trans isomerase FKBP1A; Peptidyl-prolyl cis-trans isomerase	P62942; Q5W0X3 ; A0A087 WZM5	3	3	3	41.7	41.7	41.7	11.95	31.8	1.6E+09	40	NaN	-0.8494	-0.9839	-1.7398	-1.6948	-1.9470

Table S7. Significantly enriched proteins by probe **4** compared to competition with paclitaxel **5** 25 μM, full dataset in Table S8. Related to Figure 6.

Gene names	Protein names	Majority protein IDs	Peptides	Razor + unique peptides	Unique peptides	Sequence coverage [%]	Unique + razor sequence coverage [%]	Unique sequence coverage [%]	Mol. weight [kDa]	Score	Intensity	MS/MS count	Normalised Log_2(LFQ intensity)					
													OXS008450			OXS008450/paclitaxel 25 μM		
													1	2	3	1	2	3
TUBB; TUBB2B; TUBB2A	Tubulin beta chain; Tubulin beta-2B chain; Tubulin beta-2A chain	Q5JP53; P07437; Q5ST81; Q9BVA1; Q13885	24	24	4	59.2	59.2	10.6	47.77	323	1.7E+11	833	5.7159	5.6001	5.8671	4.8092	4.6922	4.2793
SRPRB	Signal recognition particle receptor subunit beta	Q9Y5M8	7	7	7	31.4	31.4	31.4	29.7	45.9	5E+09	58	0.3632	0.2234	0.2824	-1.6565	-1.6003	-0.4379
TOMM22	Mitochondrial import receptor subunit TOM22 homolog	Q9NS69	6	6	6	59.9	59.9	59.9	15.52	245	7.8E+10	222	3.6598	4.3183	4.5909	2.8063	2.5150	2.8173
HMGCS1	Hydroxymethylglutaryl-CoA synthase, cytoplasmic	Q01581	16	16	16	38.3	38.3	38.3	57.29	265	2E+10	150	2.2660	2.6619	2.7035	1.1665	0.8726	1.3732
VDAC3	Voltage-dependent anion-selective channel protein 3	Q9Y277; E5RJN6; E5RHZ6; E5RFP6	12	12	12	50.5	50.5	50.5	30.66	105	1.6E+10	130	2.1622	2.1737	1.8625	0.6697	0.6287	0.7537
MYL6	Myosin light polypeptide 6	F8W1R7; G3V1V0; P60660; F8VPF3; J3KND3; G8JLA2; B7Z6Z4; G3V1Y7	5	5	5	41.4	41.4	41.4	16.29	29.6	4.5E+09	61	0.1486	0.2889	0.4745	-1.3897	-0.8686	-0.8626
PDIA4	Protein disulfide-isomerase A4	P13667	13	13	13	27.6	27.6	27.6	72.93	38.5	2.9E+09	70	-0.7640	-0.1033	-0.2837	-1.7861	-1.9867	-1.2968
TUBB4B; TUBB4A	Tubulin beta-4B chain; Tubulin beta-4A chain	P68371; P04350	24	5	3	56.9	13.7	7	49.83	85.3	9.3E+09	70	1.2327	1.4241	1.7439	NaN	0.2235	0.1757

Gene names	Protein names	Majority protein IDs	Peptides	Razor + unique peptides	Unique peptides	Sequence coverage [%]	Unique + razor sequence coverage [%]	Unique sequence coverage [%]	Mol. weight [kDa]	Score	Intensity	MS/MS count	Normalised Log_2(LFQ intensity)					
													OXS008450			OXS008450/paclitaxel 25 µM		
													1	2	3	1	2	3
FLNA	Filamin-A	Q60FE5; P21333; A0A087WWY3	26	26	26	14	14	14	278.2	169	4.5E+09	108	-0.2894	0.3664	0.4094	-1.1840	-0.8355	-1.2064
ATP2A2	Sarcoplasmic/endoplasmic reticulum calcium ATPase 2	P16615; H7C5W9	18	18	12	20.4	20.4	14.5	114.8	140	1.5E+10	164	1.7657	1.7246	2.3735	0.4023	0.7017	1.0912
RTN4	Reticulon; Reticulon-4	F8W914; Q9NQC3; H7C106; A0A0U1RQR6	6	6	6	28.7	28.7	28.7	37.14	48.2	1.1E+10	75	1.0253	1.0080	1.0051	0.0463	-0.0916	NaN
DCTPP1	dCTP pyrophosphatase 1	Q9H773	5	5	5	39.4	39.4	39.4	18.68	78.8	5.2E+09	61	1.0178	0.7472	0.7494	-0.5277	-0.2035	0.1728
HNRNPL	Heterogeneous nuclear ribonucleoprotein L	P14866; M0QXS5; A0A3B3ITJ4	9	9	6	21.6	21.6	14.3	64.13	56	4.2E+09	59	0.0432	0.2138	0.5863	-0.7971	-0.9151	-0.5133
TRIM28	Transcription intermediary factor 1-beta	Q13263; M0R0K9	14	14	14	30.8	30.8	30.8	88.55	257	9.6E+09	108	1.4484	1.4414	1.5596	0.4710	0.2544	0.7821
MYADM	Myeloid-associated differentiation marker	C9JJV6; C9JZL8; C9J5M0; C9JC07; Q96S97	2	2	2	22.6	22.6	22.6	15.86	28.7	6.7E+09	49	0.9750	0.8764	0.5115	-0.3719	-0.1499	0.1951
IMMT	MICOS complex subunit MIC60	B9A067; Q16891; C9J406; H7C463	18	18	18	30.5	30.5	30.5	78.97	97.2	1E+10	111	0.7136	0.9082	1.0688	0.1639	-0.2118	0.2452
EEF1G	Elongation factor 1-gamma	P26641	9	9	9	24.3	24.3	24.3	50.12	90	1.1E+10	111	1.5808	1.4077	1.2721	0.3163	0.7347	0.7340
